# Neurocomputational mechanism of controllability inference under a multi-agent setting

**DOI:** 10.1371/journal.pcbi.1009549

**Published:** 2021-11-09

**Authors:** Jaejoong Kim, Sang Wan Lee, Seokho Yoon, Haeorm Park, Bumseok Jeong

**Affiliations:** 1 Graduate School of Medical Science and Engineering, Korea Advanced Institute for Science and Technology (KAIST), Daejeon, Republic of Korea; 2 Division of the Humanities and Social Sciences, California Institute of Technology, Pasadena, California, United States of America; 3 KAIST Institute for Health Science and Technology and KI for Artificial Intelligence, KAIST, Daejeon, Republic of Korea; 4 Department of Bio and Brain Engineering, Korea Advanced Institute of Science Technology (KAIST), Daejeon, Republic of Korea; 5 Program of Brain and Cognitive Engineering, Korea Advanced Institute of Science Technology (KAIST), Daejeon, Republic of Korea; 6 KAIST Institute for Artificial Intelligence, Korea Advanced Institute of Science Technology (KAIST), Daejeon, Republic of Korea; 7 KAIST Center for Neuroscience-inspired AI, Korea Advanced Institute of Science Technology (KAIST), Daejeon, Republic of Korea; 8 Yeongnam University Hospital, Daegu, Republic of Korea; University of Tokyo: Tokyo Daigaku, JAPAN

## Abstract

Controllability perception significantly influences motivated behavior and emotion and requires an estimation of one’s influence on an environment. Previous studies have shown that an agent can infer controllability by observing contingency between one’s own action and outcome if there are no other outcome-relevant agents in an environment. However, if there are multiple agents who can influence the outcome, estimation of one’s genuine controllability requires exclusion of other agents’ possible influence. Here, we first investigated a computational and neural mechanism of controllability inference in a multi-agent setting. Our novel multi-agent Bayesian controllability inference model showed that other people’s action-outcome contingency information is integrated with one’s own action-outcome contingency to infer controllability, which can be explained as a Bayesian inference. Model-based functional MRI analyses showed that multi-agent Bayesian controllability inference recruits the temporoparietal junction (TPJ) and striatum. Then, this inferred controllability information was leveraged to increase motivated behavior in the vmPFC. These results generalize the previously known role of the striatum and vmPFC in single-agent controllability to multi-agent controllability, and this generalized role requires the TPJ in addition to the striatum of single-agent controllability to integrate both self- and other-related information. Finally, we identified an innate positive bias toward the self during the multi-agent controllability inference, which facilitated behavioral adaptation under volatile controllability. Furthermore, low positive bias and high negative bias were associated with increased daily feelings of guilt. Our results provide a mechanism of how our sense of controllability fluctuates due to other people in our lives, which might be related to social learned helplessness and depression.

## Author summary

How we perceive controllability over an outcome if there are multiple other agents who can simultaneously influence that outcome? Previous ‘single-agent’ studies showed that an agents’ inferred controllability depends on contingency between its own action and following outcome and this inference involves striatum. Here, we show that in a multi-agent setting, other people’s action-outcome contingency information is integrated with one’s own action-outcome contingency to infer controllability, which was explained as a biased Bayesian inference. Notably, bias in inference played an adaptive role under volatile controllability and was associated with a perception of guilt. Striatum and temporoparietal junction (TPJ) were involved in this multi-agent Bayesian controllability inference and this controllability information was leveraged to increase motivated behavior in the vmPFC. Our results first provide a neurocomputational mechanism of multi-agent controllability inference.

## Introduction

Through interactions with the surrounding world, humans learn about things that they can influence though their own actions as well as things they cannot change through their behavior. Perceived controllability is a belief that one can control the outcome of a specific situation through their behavior [1] and plays an important role in how we behave and feel [2]^,^ which can be seen, for example, in learned helplessness theory [3]. Classically, controllability has been thought to be estimated or inferred from the learned relationship between one’s own action toward the environment and following contingent outcomes [4]. We perceive a situation as controllable if our action and outcome are correlated in which the correlation between an action and an outcome is computed retrospectively by using previous action-outcome history [5]. We will call this kind of controllability inference ‘single-agent controllability inference’, meaning that there is only one agent that interacts with the environment during decision making. Then, if people perceive the world as controllable, they are motivated to exhibit more proactive and cognitively demanding strategies because they expect that the outcome depends on their behavior, while low controllability promotes reactive or helplessness behavior because in the uncontrollable situation [1,3,4]. Animal studies and neuroimaging studies have shown that single-agent controllability inference and its influence on decision making involve a corticostriatal dopaminergic system such that the striatum detects the controllable situation and the ventromedial prefrontal cortex (vmPFC) modulates behavior according to controllability [3,4].

However, unlike the situation in the single-agent controllability inference, the usual decision-making situation in our real life involves multiple agents who can influence the decision outcome, including myself and other people. In this case, to identify whether I have controllability over the outcome or not, we have to exclude the possibility that other people except myself might have controllability by observing other people. We will call this kind of controllability inference ‘multi-agent controllability inference’ in contrast to the single-agent controllability inference. Considering classical studies showing the influence of controllability in various aspects of our behavior and emotion, establishing a mechanism of multi-agent controllability inference is important because it is much more common than single-agent controllability inference. However, no previous studies have investigated computational models of multi-agent controllability inference and how the brain implements this inference process. Therefore, in this study, we investigated these questions using the novel multi-agent Bayesian controllability inference (MABC) model and model-based fMRI analyses.

Based on previous studies, we formulated the following hypotheses. First, we expected that to infer multi-agent controllability, people might have to leverage other people’s action-outcome correlation information in addition to one’s own action-outcome correlation information. If so, our next question would be the computational mechanism of integrating these multiagents’ (both self and others’) behavioral information. Similar to the Bayesian integration model of multiple sensory information [6], we expected that the multi-agent controllability inference would be explained as a Bayesian inference of posterior controllability given prior and the multiple agents’ action-outcome observations. As a possible candidate neural mechanism, we expected that multi-agent controllability inference would recruit corticostriatal circuits, meaning the generalization of these regions’ roles from single-agent controllability to multi-agent controllability. Furthermore, considering that integrating others’ action-outcome information might be crucial in multi-agent controllability, we hypothesized that recruitment of regions previously known for processing others’ behavioral information might be necessary, such as the temporoparietal junction (TPJ). This region is known for theory of mind [7] and integrating others’ dissent information during decision making [8]. Finally, as in previous single-agent studies, we expected that this inferred multi-agent controllability would also facilitate motivated behavior and influence emotion. In particular, considering that utilizing value is cognitively costly (especially when values of actions are similar) [9], we expected that a motivation to utilize value would be increased by multi-agent controllability and that the emotion that arises from social situations, such as guilt, would be affected by controllability.

**Fig 1:**
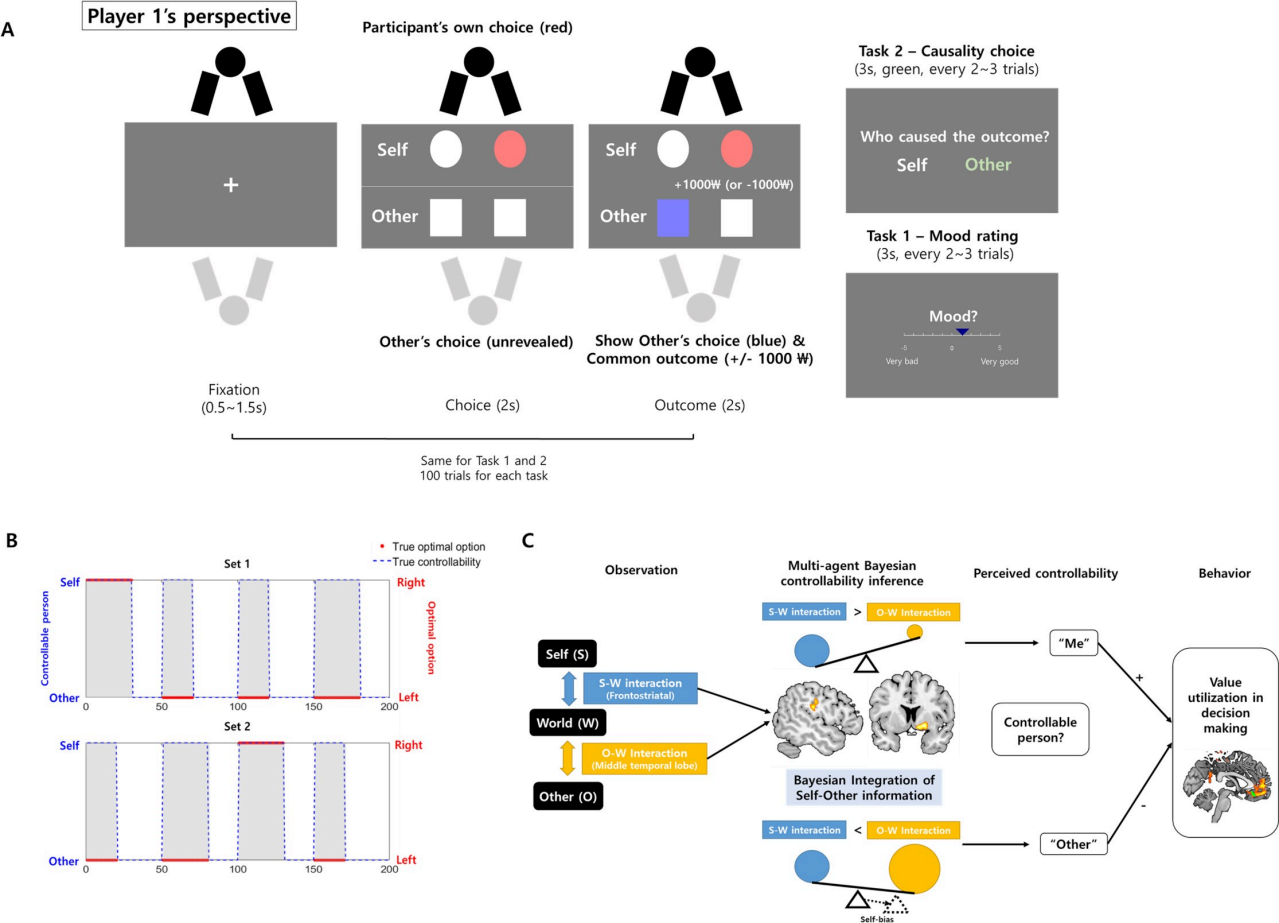
Experimental paradigm and graphical summary. **(A) Two-person decision-making task with changing controllability.** Two participants simultaneously performed the task in a separate room. Although they expected to be playing with each other, they actually played with an artificial reinforcement learning agent. Participants were instructed to perform the two-alternative choice task such that each person would make a choice separately and the resulting outcome (either reward or loss) would be the only common element for them. Participants were told that in every trial, only one person among them had control over the outcome (“controller” or “person in control”), while another person’s choice would not influence the outcome (“person without controllability”), and the controller could change after some trials. There was always an optimal option, but only for the controller, that resulted in reward with high probability (80% reward/20% loss, actual probability not known to participant) and a suboptimal option that resulted in loss with high probability (80% loss/20% reward); there was no optimal option for the person not in control. Therefore, the choice made by the controller was important for both of them, while the choice made by the person not in control did not matter. The controller was switched every 20–30 trials. To assess perceived controllability, participants were asked who caused the outcome every two or three trials (causality choice, Task 2), and they were also asked to rate their current mood every two or three trials (Task 1). **(B) True controllability structure and the location of optimal choices in self-controllable blocks.** The gray shaded area represents the self-controllable block. The optimal option only existed in self-controllable blocks. Note that in this figure, task 1 (100 trials) and task 2 (100 trials) were concatenated for visualization (1–100 trials in this figure are task 1 and 101–200 trials are task 2). **(C) Conceptual summary of the experiment.** An agent observed two kinds of interactions–an interaction between self and environment and the interaction between other person and environment. The TPJ and striatum integrate both types of information to infer multi-agent controllability. Then, inferred controllability information was utilized to influence motivated behavior in the vmPFC.

To test these hypotheses, we designed a novel task involving two participants (N = 103, Fig 1A, *Materials and Methods*) in which only one participant had control over the outcome (reward or loss), meaning that only one person’s behavior can influence the outcome. The controller or person in control changed every 20–30 trials throughout the task, and the person who had true controllability was not revealed to the participants; thus, the participants had to infer the true controllability structure using the action-outcome history of both the participants themselves and the other person. Note that from the perspective of one participant (e.g., player 1; among two participants), blocks of trials in which a participant had true controllability were called “self-controllable” blocks, and the blocks of trials in which another person had controllability were called “other-controllable” blocks. Participants were occasionally asked about who would be in control in the current context (causality choice, Fig 1) or asked about their mood. Behavioral analyses, including computational modeling and functional MRI analyses (fMRI, N = 30), based on our computational model were performed.

## Results

### Behavioral effect of others’ action-outcome contingency on controllability perception

Participants correctly identified who controlled the situation (true controllability) significantly better than chance (mean controllability accuracy (sd): 59.5% (10); t[9.70] = 102.00, p<0.0001, CIs: 0.076 to 0.115 in one-sample t-test testing whether difference between accuracy and chance level is above 0).

**Fig 2:**
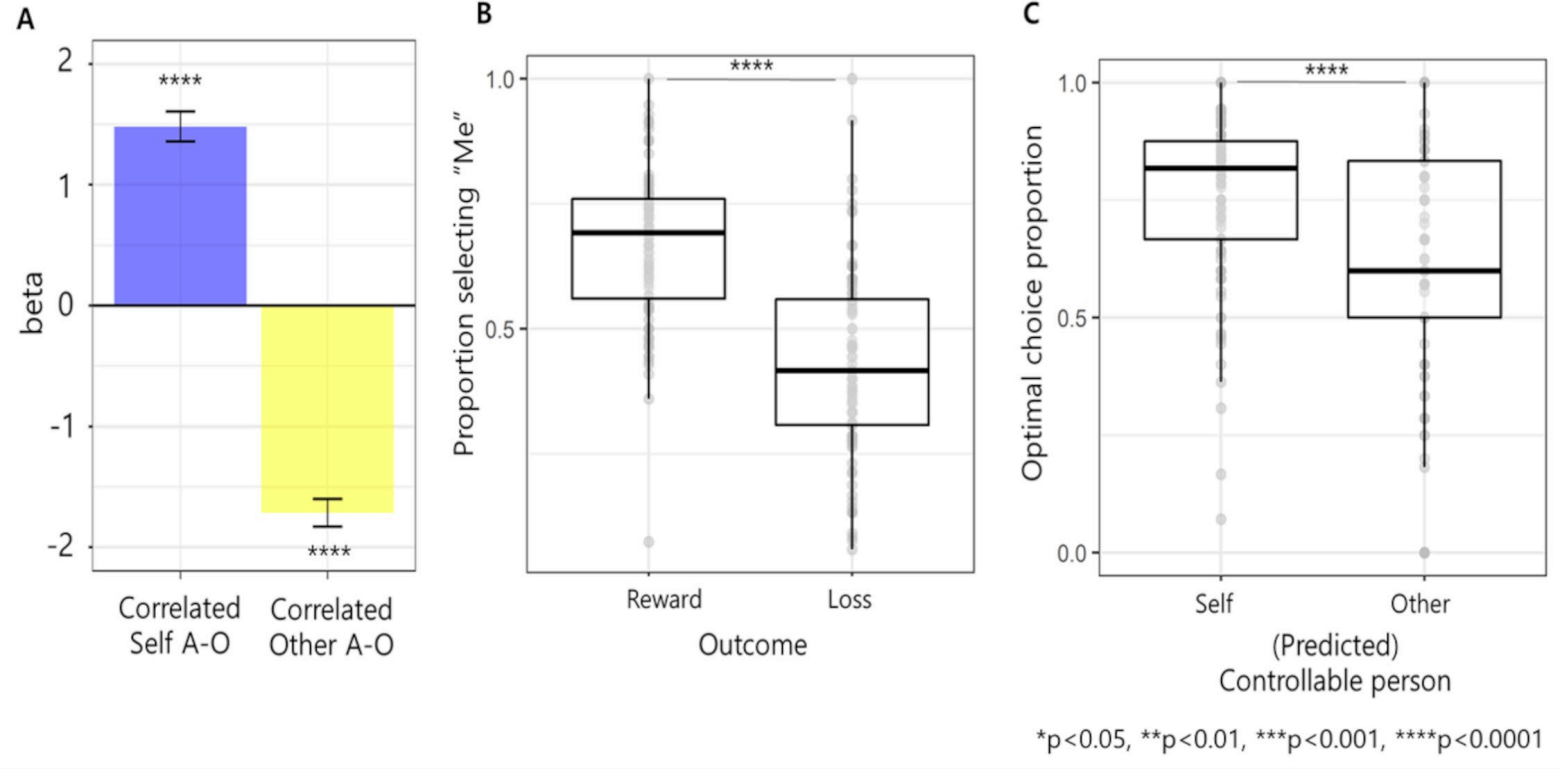
Behavioral analysis results. **(A) Opposite effect of correlated self- and others’ action outcomes on perceived controllability.** Mixed-effect logistic regression showed that correlated action-outcome of oneself (CIx_self_ = 1) significantly increased the probability of perceiving oneself as controller, while correlated action-outcome of other person (CIx_other = 1_) significantly decreased this probability, meaning that both action-outcome information is utilized in multiagent controllability inference. A-O in this figure represents action outcome. **(B) Difference in perceived controllability between reward and loss.** The proportion of trials perceived as self-controllable was higher after reward than after loss. **(C) Effect of perceived controllability on optimal choice.** The proportion of optimal choices was higher in the trials after participants selected that they had caused the outcome than after selecting other persons who had caused the outcome.

Then, we expected that the correlated relationship between participants’ own action and outcome would increase perceived controllability, while the correlated action and outcome of other persons would decrease perceived controllability. Here, a correlated relationship means that one’s specific action (e.g., left) is consistently associated with the same outcome (reward), while the other action (right) is consistently associated with another outcome (loss). The correlation index (CIx) of each trial was defined such that CIx = 1 at trial k means that a participant showed correlated action-outcome for two consecutive trials (trial k-1 and k), whereas CIx = 0 means the opposite (*Materials and Methods*). Both CIx of self (CIx_self_) and other person (CIx_other_) was calculated in every trial. By using a mixed-effect logistic regression, we tested the fixed effect of CIx_self_, CIx_other_ and their interaction on participants’ perception as a controller (selecting “Self” in the causality choice). Consistent with previous studies, we found that observing correlated own action and outcome increased the probability of perceiving oneself as controllable (beta = 1.48, t[4080] = 11.91, p<0.0001, Fig 2A). Importantly, however, observing correlated others’ action and outcome decreased the probability of perceiving oneself as controllable (beta = -1.78, t[4080] = -15.04, p<0.0001, Fig 2A), showing that others’ behavioral information is utilized during controllability inference in addition to their own behavioral information. The interaction effect was not significant (beta = 0.29, t[4080] = 1.86, p = 0.0626). Note that the correlation between CIx_self_ and CIx_other_ did not reach statistical significance (Pearson r_partial_ = -0.00, p = 0.866).

Furthermore, we found that reward biases people to perceive them as a more controller (t[102] = 9.14, p<0.0001, CIs: 0.185 to 0.287, d = 0.901, paired t-test comparing the proportion of selecting oneself as a controller between reward and loss trials preceding causality rating trials, Fig 2B). Therefore, we added reward and its interaction with both CIx as a fixed effect in previous mixed effect logistic regression, which not only showed a similar effect of CIx_self_ and CIx_other_ (positive and negative, respectively; all p<0.0001) but also showed a positive interaction between reward and CIx_self_ (beta = 0.96, t[4077] = 5.27, p<0.0001), meaning that reward amplifies CIx_self_’s effect on controllability perception (or inference).

Finally, considering the effect of controllability on motivation, we expected that when participants perceived themselves as a controller, their motivation to make better choices would increase optimal choices. Indeed, participants were more likely to make optimal choices when they believed they had control (compared optimal choice proportion between trials right after selecting “Self” in the causality choice and trials right after selecting “Other” in the causality choice using paired t-test, t[102] = 4.88, p<0.0001, CIs: 0.078 to 0.184, d = 0.481, Fig 2C). Here, the optimal choice was defined as choosing an option that was designed to have a higher probability of reward than the other option when one had true controllability. Therefore, this optimal choice can be defined only within self-controllable blocks. Additionally, by considering the possibility that participants might have not learned which option is optimal in early trials of self-controllable blocks, we performed the same analysis only using the late self-controllable block (final 10 trials of self-controllable blocks), which showed a consistent result as the previous one (t[85] = 2.24, p = 0.0280, CIs: 0.011 to 0.186, d = 0.260, paired t-test).

### Multiagent Bayesian controllability inference model

**Fig 3:**
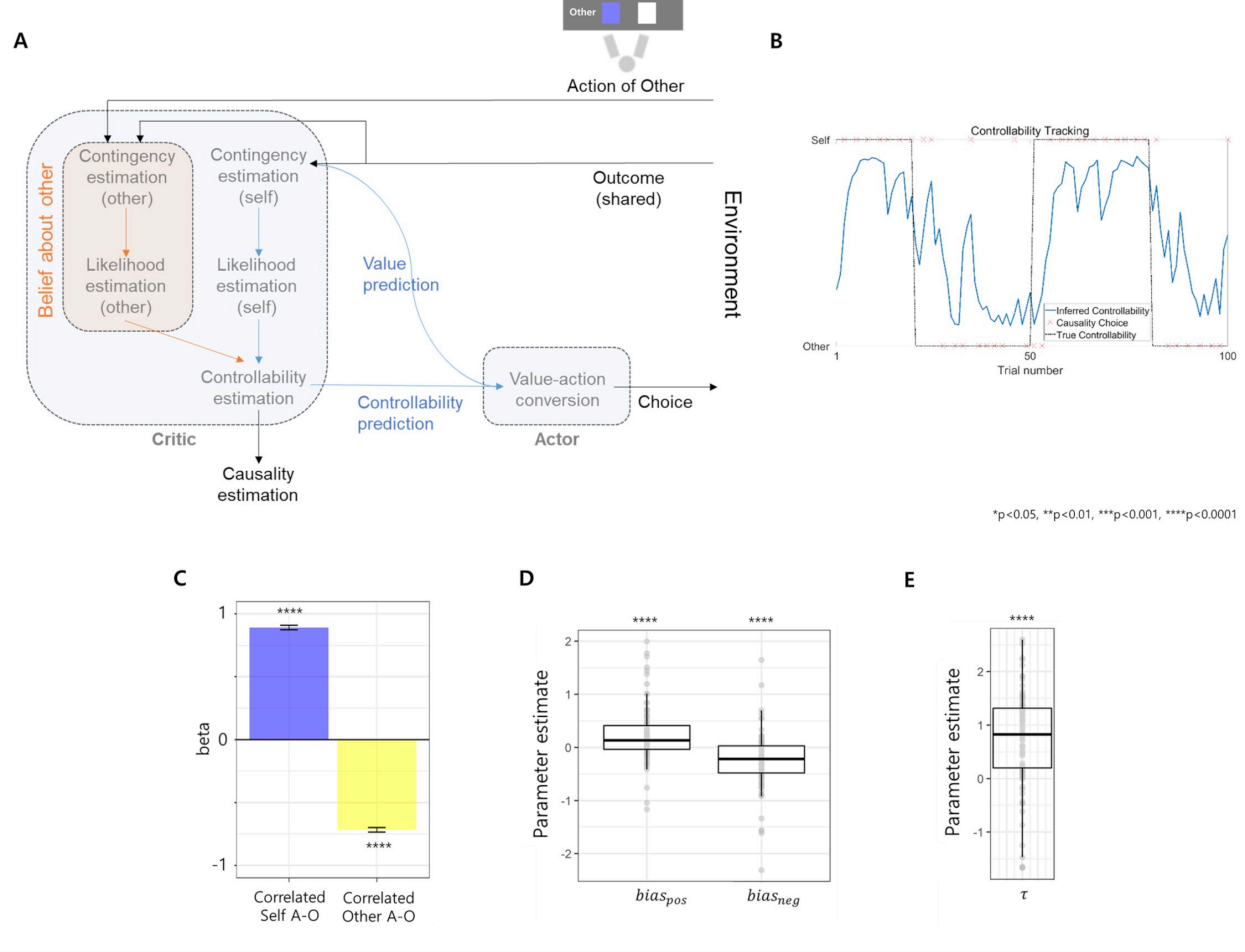
Computational model of multi-agent controllability inference. **(A) Schematic description.** Action of self or action of another person caused an outcome according to the true controllability. Then, an agent obtains observations including outcome, own action and other’s action. In the multi-agent controllability inference module, the agent computes self-likelihood and other-likelihood using this information. Finally, both likelihoods are integrated in a Bayesian fashion with bias to estimate multi-agent controllability. In our task, this estimated multi-agent controllability was utilized not only to make causality choices but also in the next trial as a predicted controllability, which increased value utilization during the value-action conversion to make value-dependent choices. **(B) Example trajectory of the inferred controllability.** This plot shows the example trajectory of the controllability (blue line) extracted from the winning MABC model. Red “x” is the participant’s causality choice, and the black line is the true controllability. **(C) Effect of correlated action outcome on inferred controllability.** Similar to the behavior analysis, correlated own action-outcome increased inferred multi-agent controllability, while correlated others’ action-outcome decreased it. **(D) Bias of the controllability inference.** Both biases were significant such that *bias*_*pos*_>0 while *bias*_*neg*_<0, meaning that people tend to prioritize self-likelihood during multi-agent controllability inference after reward, which increases perceived controllability, while the opposite tendency appears after loss. (E**) Controllability-dependent value utilization.** τ was larger than zero, meaning that the predicted controllability increases the value utilization during decision making.

Behavior analyses suggest that observations regarding the action-outcome relationship for both oneself and others are utilized for controllability inference. We derived a controllability inference under a multi-agent setting as a Bayesian inference of posterior controllability given both the observed self- and other’s action-outcome history and the prior, which is similar to the multisensory integration model (see *S1 Text* for posterior derivation). We also showed that although our task situation involves only two agents, this model can be applied to a situation involving three or more people (see *S1 Text* for this generalization). A key aspect of this multi-agent Bayesian controllability inference model (MABC model) is that an agent’s posterior controllability is determined by the ratio between the likelihood of the current outcome given one’s own action (self-likelihood; e.g., “how likely is it that my choice has resulted in the current reward if I assume that I was in control?) and the likelihood of the current outcome given other people’s action (other-likelihood; e.g., “how likely is it that other people’s choice resulted in the current reward if I assume that the other person was in control?”) such that a relative increase in self-likelihood increases the inferred controllability (Fig 3A and 3B). These likelihoods can be represented by

$${likelihood}_{self}=p(outcome=reward|my\;action=left,\;controller=self)$$ (1)

and

$${likelihood}_{other}=p(outcome=reward|other’s\;action=right,\;controller=other)$$ (2)

respectively. These likelihoods were computed using the learned action-outcome relationship (p(outcome|action)) in which learning was based on the classical static-learning rate reinforcement learning rule [10].

Then, they were combined to make posterior controllability inferences as follows:

$${Posterior}_{self}=\frac{{likelihood}_{self}*{prior}_{self}}{{likelihood}_{self}*{prior}_{self}+{likelihood}_{other}*{prior}_{other}}$$ (3)

The above equation shows that the posterior inferred controllability increases as the relative likelihood ($\frac{{likelihood}_{self}}{{likelihood}_{other}}$) increases. A detailed description of the MABC model can be found in the *Materials and Methods*. Each likelihood computation was accomplished by using a learned action-outcome relationship (Fig 3A and 3B).

Using this model as our core controllability inference hypothesis, we constructed thirty-one computational models that include models that do not consider others’ behavior (S2A Fig). The winning model was the MABC model with reward biases and controllability-guided value utilization (protected exceedance probability (PEP) = 1, *Materials and Methods*). This model successfully replicated the effect of CIx_self_ and CIx_other_ on perceived controllability in our previous behavioral analyses (Fig 3C and *S1 Text*). Bias parameters represent the degree of relative weight on self-likelihood compared to other-likelihood during multi-agent controllability inference. A bias parameter greater than 0 means that they would overestimate their own controllability by overweighing the self-likelihood compared to other-likelihood and vice versa. *bias*_*pos*_ is the bias after reward, and *bias*_*neg*_ is the bias after loss. Consistent with the behavior analysis results, participants’ inferred controllability was overestimated after reward by overweighing self-likelihood (t[102] = 4.47, p<0.0001, CIs: 0.126 to 0.326, d = 0.441, one-sample t-test of *bias*_*pos*_, Fig 3D) while underweighting self-likelihood when the outcome was loss (t[102] = -5.03, p<0.0001, CIs: -0.357 to -0.155, d = -0.495, one-sample t-test of *bias*_*neg*_, Fig 3D).

Furthermore, in a winning MABC model, high inferred controllability of the previous trial (predicted controllability) increased value utilization, meaning an increased deterministic tendency of choosing an option with high subjective value. More specifically, predicted controllability increased the inverse temperature of the decision to increase value utilization, and the degree of inverse temperature modulation by predicted controllability was parametrized by τ (*τ*>0 means that predicted controllability increases the inverse temperature and vice versa; note that *τ* is not an inverse temperature itself but its modulator, *Materials and Methods*). τ was significantly above 0 in the one-sample t-test, meaning that predicted controllability increased value utilization (t[102.00] = 8.66, p<0.0001, CIs: 0.568 to 0.906, d = 0.854, Fig 3E).

### Reward-driven controllability overestimation bias improves adaptability at the cost of illusory control

**Fig 4:**
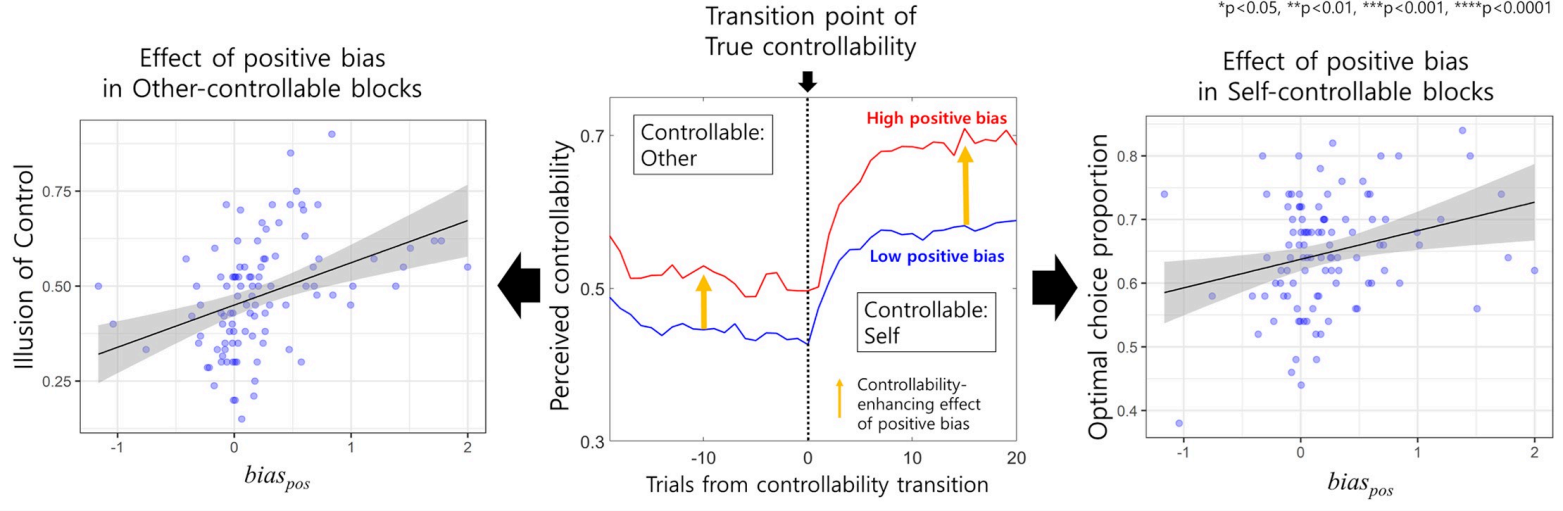
Effect of reward-driven positive inferential bias. Participants with high positive bias exhibited a higher level of predicted controllability both in other-controllable blocks and self-controllable blocks compared to the level of predicted controllability in participants with low positive bias (middle). Although such bias induced an illusion of control in other-controllable blocks (left), it helped participants to rapidly regain controllability in self-controllable blocks (middle) and increased decision optimality in those blocks (right).

Essentially, overestimation bias might cause the illusion of control in an uncontrollable environment, ultimately wasting one’s effort. Indeed, *bias*_*pos*_ was positively correlated with the illusion of control (proportion of selecting “Self” in the causality choice when the true controller was the other, Pearson r_partial_ = 0.39; p<0.0001, Fig 4, left), while negative bias was not (p>0.5; see *S1 Text* for more explanation). Note that all correlation analyses we performed were partial correlations using sex as a covariate. Then, what is the advantage of this bias? We expected that in an environment where the controller often changes, positive bias might facilitate a fast recognition of change from an uncontrollable environment to a controllable environment, leading to an increased optimal choice by value utilization.

Consistent with our expectation, positive bias was significantly correlated with choice optimality in the second half of the self-controllable block (Pearson r_partial_ = 0.26; p = 0.0080, Fig 4, right), while the first half was not (Pearson r_partial_ = -0.18; p = 0.0648), showing that a controllability-induced value-dependent decision only influences the optimal choice when one has sufficiently learned about the value of options. Additionally, a negative bias was not related to choice optimality (p>0.05 for both the first and second halves). These results show that although positive bias increases the illusion of control when another person has true controllability, it improves adaptability by promoting fast recognition of the return of true controllability.

### Beneficial effect of bias on momentary emotion and guilt tendency

Inferred controllability not only influenced decision making but also amplified outcome-related emotion such that positive feeling after reward and negative feeling after loss were increased proportionally to controllability (S4B Fig and *S1 Text*). Therefore, bias had another beneficial effect of increasing positive feelings after reward and decreasing negative feelings after loss during our experimental task. Furthermore, considering that people prone to feeling guilty tend to think that they have caused the negative outcome, even in a situation in which they were not actually in control, which is similar to the negative bias in our MABC model. We found that people prone to guilt in their daily life (measured by Test of Self-Conscious Affect 3 (TOSCA-3) score, *S1 Text*) tend to overestimate their control of a situation resulting in loss (less negative *bias*_*neg*_, Pearson r_partial_ = 0.27; p = 0.0056, S4C Fig). Furthermore, these people also underestimated their ability to control a situation after reward (less positive *bias*_*pos*_, Pearson r_partial_ = -0.25; p = 0.0129).

### Neural substrate of multi-agent controllability estimation

**Fig 5:**
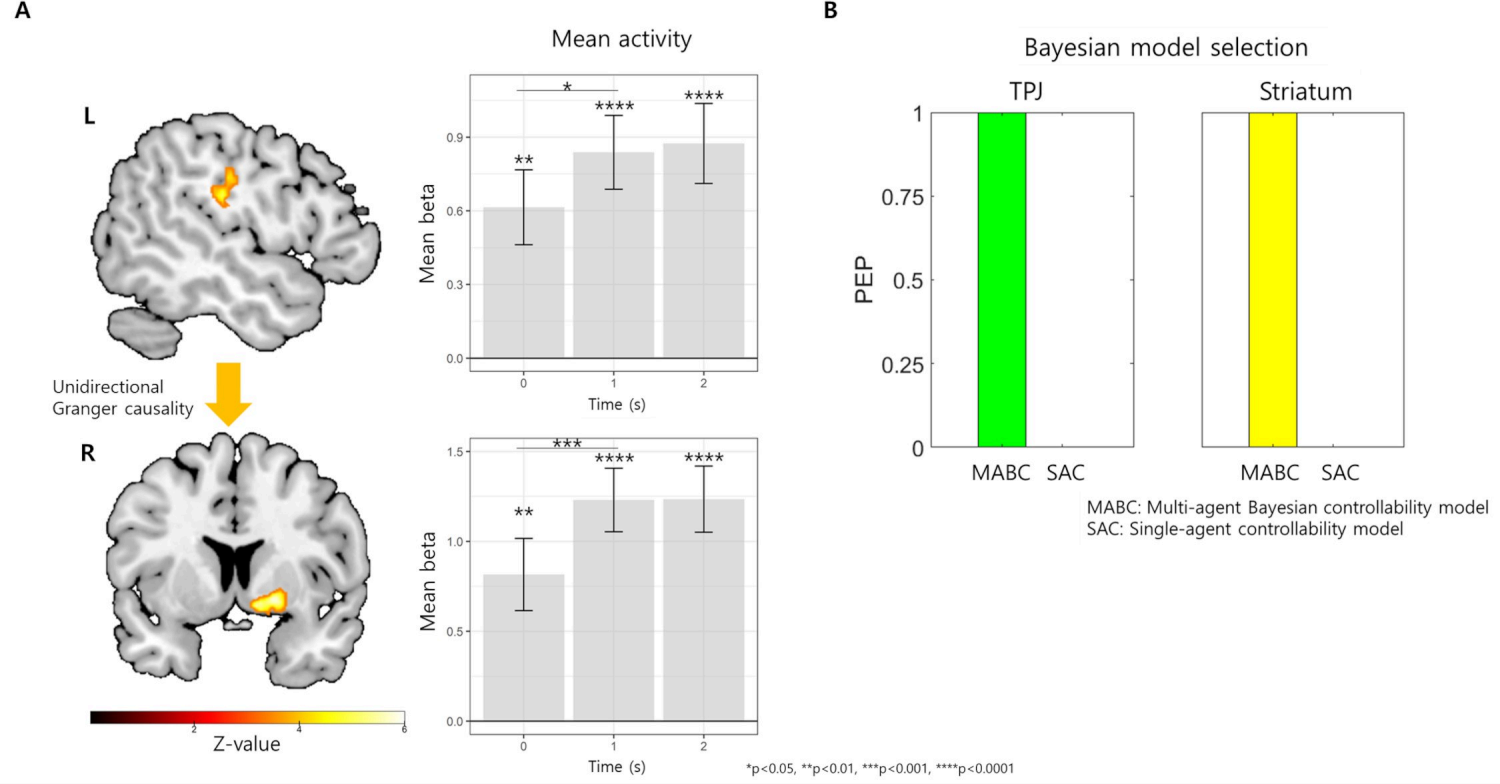
Neural mechanism of multi-agent controllability inference. **(A) Regions involved in multi-agent controllability inferences.** The BOLD signals of the right striatum (lower left) and left TPJ (right left) covaried with the inferred controllability estimated from the winning MABC model. Both regions’ controllability inference-related signals increased from 0 s to 1 s after the outcome (bar graphs with gray color). There was a unidirectional Granger causality between two regions (yellow arrow) **(B) Bayesian model selection results.** Bayesian model selection showed that the MABC-based neural model explains the activity of both regions much better than a single-agent controllability model (PEP = 1 for the MABC-based model in both regions), meaning that these regions are related to utilizing multiagent information rather than single-agent information only.

In the model-based fMRI analyses using log-odds of inferred multi-agent controllability (log-controllability ratio, LCR in short) estimated from the winning MABC model as a parametric modulator (GLM1, see *Materials and Methods*), we found activity of the left TPJ, [–50, –22, 26], cluster-level FWE-corrected p = 0.032, Z-value of the peak voxel = 4.37, Fig 5A and S2 Table) and the right striatum ([18, 8, –6], cluster-level FWE-corrected p<0.001, Z-value of the peak voxel = 4.82, Fig 5A). These controllability signals were greater at 1 s after the outcome presentation compared to the time right after the outcome (0 s) (t[29.00] = 3.84, p = 0.0006, CIs: 0.194 to 0.635, d = 0.702, for the right striatum and t[29.00] = 2.66, p = 0.0125, CIs: 0.052 to 0.395, d = 0.486, for the left TPJ, in paired t-tests between 0 s and 1 s after outcome, Fig 5A).

In the following region of interest (ROI) analyses, we further found unidirectional Granger causality between these regions (from TPJ to striatum, S7 Fig and *S1 Text*).

### TPJ and striatum leverage other action-outcome information to estimate multi-agent controllability

We then tested whether both the TPJ and striatum perform a multi-agent controllability inference by integrating both self- and other-related information or whether their activity could be explained solely based on self-related information, meaning that these regions perform single-agent controllability inference. We compared log model evidence of two competing neural models in each region using Bayesian model selection [11]. The first model explained neural activity using the MABC model (that is, GLM1 model), while the other model explained neural activity using the computational model that only considers an effect of one’s own action and outcome (GLM2 model; inferred single-agent controllability was estimated from model 4 in S2A Fig, see also *Materials and Methods*). Bayesian model selection showed that the MABC-based neural model explains the activity of both regions much better than a single-agent model (PEP = 1 for the MABC-based model in both regions, Fig 5B).

These results show that the TPJ and striatum perform multi-agent controllability inference by leveraging other related information in addition to one’s own. In particular, one can see that the striatum, the region that plays an important role in the single-agent controllability inference, also plays an important role in multi-agent controllability inferences.

### Regions encoding other-likelihood and self-likelihood

We showed that the TPJ and striatum are involved in computing multi-agent controllability by integrating self- and other’s information. Then, which regions are involved in the computation of self-likelihood and other-likelihood? We performed GLM3 using self-likelihood and other-likelihood as parametric modulators and found that right middle temporal gyrus (MTG) activity responded to a decrease in other-likelihood ([56, –22, –8], cluster-level FWE-corrected p = 0.035, Z-value of the peak voxel = 4.53, S5 Fig and S3 Table). Considering that other-likelihood negatively influences controllability, the brain might detect a decrease in other-likelihood rather than encode it directly. On the other hand, self-likelihood activated regions known for inferring single-agent controllability and related motivated behavior, such as the vmPFC, ventral striatum (more medial to the multi-agent controllability striatum) and hippocampus (S6 Fig and S3 Table).

### Controllability estimation improves value utilization in the vmPFC

The winning MABC model showed that the predicted controllability of the next trial improves value utilization. Basically, people choose more deterministically the option with higher subjective value when the value difference (VD) between the high-valued option and low-valued option is high. Improving value utilization means that the effect of VD on deterministic decisions would be increased by the predicted controllability, meaning a positive interaction between VD and predicted controllability. At the neural level, we expected that this effect would be represented as a similar positive interaction effect, meaning an increase in the VD signal by the predicted controllability.

**Fig 6:**
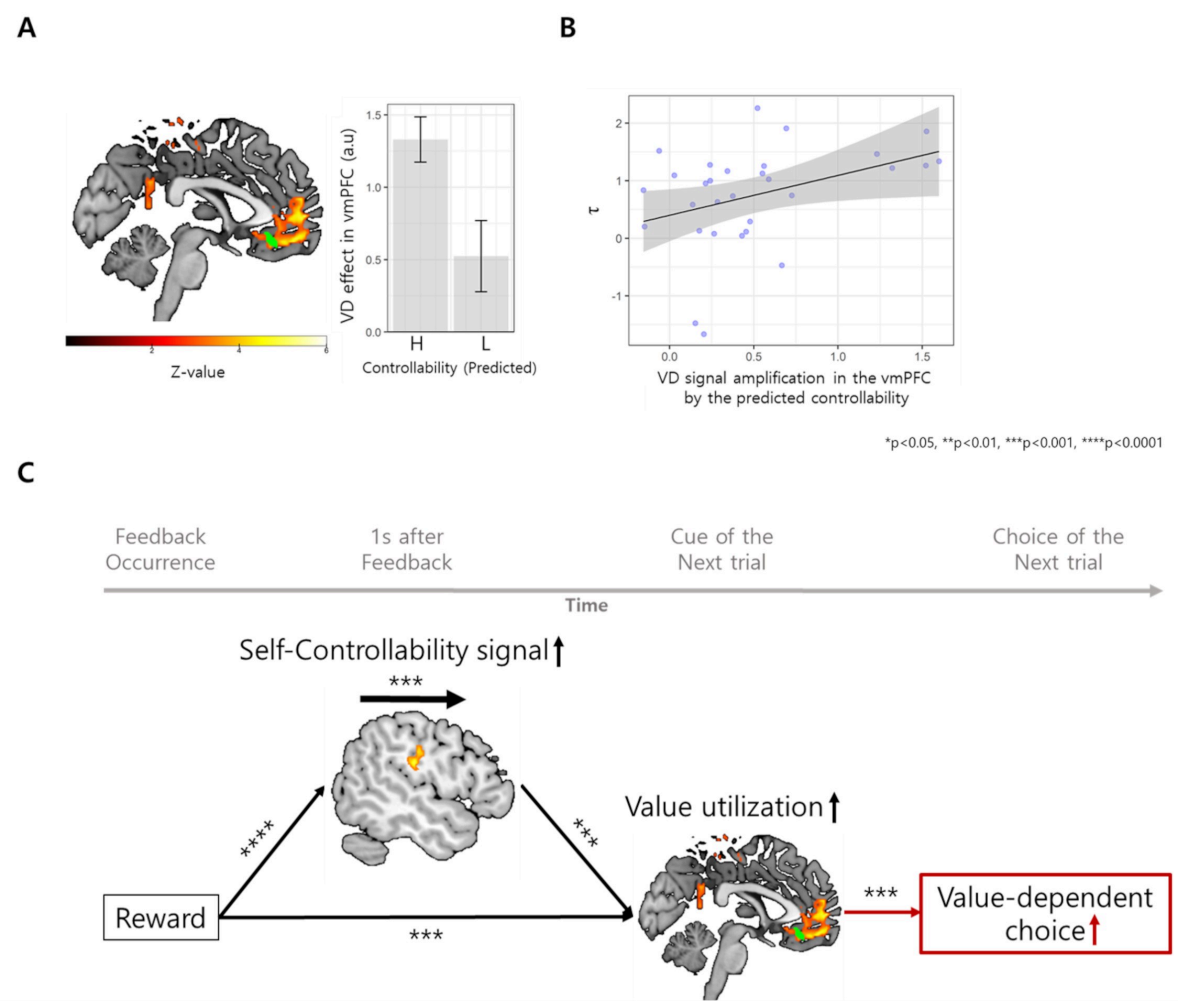
Neural mechanism of controllability-dependent policy change and reward-driven adaptability. **(A) Region increasing value utilization in response to increased multi-agent controllability.** The BOLD signal in the vmPFC covaried with VD (red and yellow). Furthermore, small regional activity within the vmPFC (green) exhibited a positive interaction effect between predicted controllability and VD, meaning that this region’s VD-related activity was amplified by the predicted controllability. **(B) Relationship between VD-related activity amplification and controllability-dependent policy modulation.** The mean beta of the positive interaction effect within the vmPFC, which is the degree of VD signal amplification by the predicted controllability, was positively correlated with the parameter τ of the winning MABC model. **(C) Neural mechanism of reward-driven policy change mediated by controllability bias.** Mediation analysis results showed that the controllability inference signal in both the TPJ and striatum mediates the effect of reward (0 s after the outcome) on the vmPFC signal of the next trial, which was involved in value utilization. This was further supplemented by the fact that vmPFC signals increased actual value-dependent choices (red arrow). Note that black solid arrows represent significant relationships in the mediation analysis, and the red solid arrow represents significant relationships in logistic regression predicting value-dependent choice by vmPFC signal.

In GLM1, parametric modulators, including VD, predicted controllability and their interaction, were locked at the cue presentation time (*Materials and Methods*). We identified that the value utilization region that encodes VD involves the vmPFC ([0, 52, –12], cluster-level FWE-corrected p<0.001, Z-value of the peak voxel = 5.18, red-yellow area in Fig 6A and S2 Table), which signal predicted participants’ value-dependent choice (choosing option with higher value, beta = 0.15, t[5973] = 4.51, p<0.0001, in the mixed-effect logistic regression, Fig 6C). Importantly, predictive controllability increased this VD signal in the vmPFC ([–2, 34, –14], small-volume corrected for the vmPFC cluster involved in VD, p = 0.012, Z-value of the peak voxel = 4.01, green area in Fig 6A and S2 Table), and this interaction effect was correlated with the value-utilization parameter (*τ*, Pearson r_partial_ = 0.38, p = 0.0441, Fig 6B), suggesting that controllability increases value utilization by enhancing the value difference signal in the vmPFC.

### The TPJ and striatum mediate the effect of positive bias on adaptive behavior under uncertain true controllability

In the winning MABC model, positive bias improved adaptability by fast recognition of true controllability, which increased value utilization. Similarly, we expected that reward would increase the controllability signal in the TPJ and striatum to increase the value difference signal in the vmPFC. We tested this hypothesis by using multilevel mediation analyses (*S1 Text*) [12]. The results showed that 1) reward increased the activity of TPJ and striatum (TPJ: mean(sd) = 0.13(0.03), p<0.0001; striatum: mean(sd) = 0.1(0.03), p = 0.0002), 2) both TPJ and striatal activity increased vmPFC activity in the next trial (TPJ: mean(sd) = 0.16(0.02), p = 0.0001; striatum: mean(sd) = 0.08(0.03), p = 0.0174), and 3) most importantly, TPJ and striatal activity mediated the effect of reward on vmPFC activity in the next trial (TPJ: mean(sd) = 0.01(0.00), p = 0.0002; striatum: mean(sd) = 0.005(0.00), p = 0.048, Fig 6C). This showed that reward-induced positive controllability bias is represented as an increased multi-agent controllability signal in the TPJ and striatum, which subsequently increases the value difference signal in the vmPFC. Additional analyses showed that this process is also related to increasing the optimal choices, but this effect was only present in the TPJ (*S1 Text*).

## Discussion

Identifying controllability becomes more complex when there are other agents who can influence the outcome compared to the single-agent setting. In this study, using the novel multi-agent Bayesian controllability model, we showed that people perform Bayesian inference to integrate multiple agents’ action-outcome profiles. MABC model-based fMRI analyses and the Bayesian model selection of neural models showed that the left TPJ and the right striatum perform multi-agent controllability inference by integrating both self- and other-likelihood information. Then, multi-agent controllability information increased motivated behavior, especially by facilitating value utilization in the vmPFC. We also found likelihood-encoding regions such that a decrease in other-likelihood was detected in the right MTG, while the self-likelihood computation involved frontostrial regions. Finally, our multi-agent controllability inference was positively biased toward the self, which increased the illusion of control. However, bias helped adaptability in the controllability-changing environment, whose effect was mediated by the multi-agent controllability inferencing areas. Furthermore, feelings of guilt in daily life were related to controllability inference bias (high negative bias and low positive bias). Therefore, in people with excessive guilt, increasing positive bias and decreasing negative bias might help reduce their guilty feelings.

An important contribution of our study is the identification of novel behavioral and neural mechanisms of inferencing controllability under a multi-agent setting. Previous single-agent controllability studies revealed that during an interaction with the environment, a correlated or deterministic relationship between an agent’s own action and outcome increases perceived controllability [4,5]. However, many decision-making situations involve multiple agents; thus, we usually consider other agents that could influence the outcome even when their existence is uncertain [13]. From the artificial intelligence perspective, recent studies of multi-agent reinforcement learning also show the importance of considering other agents because it is useful to make globally optimal decisions and resource allocation [14]. Interestingly, one recent study reported that a sense of controllability was reduced in the presence of others [15]. In that study, participants had to decide when to stop inflating a balloon to avoid a burst that resulted in a large loss. There were social trials with the presence of other persons involved in the decision-making situation (regardless of whether other persons can influence decisions or not) and nonsocial trials without other persons [15]. The authors showed that the sense of agency is reduced in social trials by mentalizing others and that the activity of mentalizing regions (e.g., the TPJ and the precuneus) was increased in social trials [15]. Although the authors suggest that the inference of controllability is influenced by the presence of others, they reported that the presence of others only “decreased” the sense of controllability because they did not consider how contingency between others’ action and outcome influences multi-agent controllability [15]. However, we showed that perceived controllability could be either increased or decreased by integrating the observed action-outcome relationship of other people. In particular, our MABC model showed that the Bayesian posterior of multi-agent controllability depends on the relative strength between self-likelihood information and other-likelihood information, meaning that one can feel low controllability even when their own action and environmental outcome is contingent if other-likelihood is high. This model is similar to the model of multisensory integration [6]. In multisensory integration, multiple sensory cues are integrated according to their precision or reliability to infer Bayesian posterior [6]. In this context, self-likelihood and other likelihood can be seen as the reliability of the controllability cue from each agent (self and other people). Another notable point is that the MABC model is not only applicable to situations with two-agent situations (self and other) but is also applicable to situations with a larger number of agents (more than two). In this case, other likelihood represents the likelihood that *any* other people except me caused the outcome (*S1 Text*). However, this generalized version of the MABC model might be unrealistic if there are too many agents (e.g., more than 10) because this model assumes that one agent simultaneously updates all other agents’ action-outcome contingency. Therefore, we should modify this model to enable realistic computation of other-likelihood in the large-sized group. For example, an attention-based learning mechanism can be applied to other-likelihood learning with an excessive number of agents [16].

From the neural mechanism perspective, we found that regions involved in single-agent controllability inference and controllability-dependent behavioral modification also perform a similar role in multi-agent controllability inferences and related behavioral change. Single-agent controllability studies have suggested that the corticostriatal circuit plays an important role [1,4] such that the striatum infers one’s controllability and the vmPFC mediates controllability-dependent behavior [3,17]. Our results generalized the role of these two regions from single-agent controllability to multi-agent controllability. In particular, MABC model-based fMRI analyses and Bayesian model selection showed that the striatum not only utilized its own action-outcome information but also integrated other people’s action-outcome information. Although this is the first study to show striatal involvement in multi-agent controllability inference, studies have shown that the striatum is involved in social comparison, such as comparing one’s reward gain with others [18], which is similar to the comparison between self-likelihood and other-likelihood in our MABC model. Furthermore, we also showed that using this multi-agent controllability information, the vmPFC increases value utilization in decision making. Interestingly, a recent study by Wang and colleagues also showed that the vmPFC is involved in the controllability’s influence on value [19], which is consistent with our result. However, in that study, controllability inflated value itself rather than increasing value utilization. Another study showed that vmPFC activity after decision outcome was modulated by internal control [2]. Those studies and our study show various ways that a vmPFC mediates the influence of controllability on decision making. In summary, our results suggest that the striatum and vmPFC play a generalized role in inferencing controllability and mediating controllability-dependent behavior in both single-agent and multi-agent settings.

The involvement of the left TPJ was the region that was not reported in previous studies of single-controllability inference. The left TPJ is necessary for reasoning about someone else’s belief [20] and has been known to be involved in understanding social intention [21]. A recent study also reported that both the left and right TPJ are involved in dissenting other people’s consensus opinions [8], and another study showed that left and right TPJ activity is involved in the mentalizing process during decision making in the presence of others [15]. These results are consistent with our result in that the left TPJ integrates other-likelihood information, which is a kind of reasoning regarding the likelihood of the current outcome from another person’s perspective.

Granger causality analysis showed predictive causality from the TPJ to the striatum, while there was no predictive causality in the reverse direction, suggesting the possibility that a multi-agent controllability inference signal in the TPJ might be predictive of a controllability inference signal in the striatum. However, because Granger causality does not tell us about the effective connectivity between regions [22], our results should be treated with great caution, and future studies might investigate their relationship using analyses such as dynamic causal modeling [23]. We speculate that controllability inference completed in these two regions might be conveyed to the vmPFC to increase value utilization.

In summary, we showed that the striatum and vmPFC are likely to act as controllability sensors and effectors, respectively, in both single-agent and multi-agent settings. However, in the multi-agent setting, an inference of controllability also involves the TPJ, and this inference within the TPJ and striatum utilizes both others’ action-outcome information and one’s own action-outcome information. The final notable point is that while the TPJ was involved in utilizing integrated self- and other-likelihood information, pure other-likelihood information was related to the right MTG, a region involved in simulating others’ minds [24].

One interesting extension that we can infer from our result regarding how multi-agent controllability inference influences behavior (value utilization) is the possible influence of the multi-agent environment on learned helplessness behavior. As the expected value of control theory suggests [25], there is no need to make deliberative and motivated behavior when one perceives no controllability over an environment. Therefore, many single-agent studies, including animal studies, have shown that if an agent’s own action does not seem to reliably change the environment, they exhibit learned helplessness behavior [3]. However, learned helplessness in humans usually occurs in multi-agent situations, such as social life, especially in highly competitive groups, and our results indicate that this social learned helplessness can be induced even in situations where environmental change highly correlates with one’s own action if another’s action-outcome correlation is higher. We expect that such social learned helplessness might involve the TPJ-striatum-vmPFC circuit, which can be investigated in future studies.

We also identified a neurocomputational mechanism regarding how reward-driven controllability overestimation bias leads to adaptive behavior under volatile true controllability. Positive bias facilitates the recognition of self-controllability when one regains true controllability and is able to make better decisions by increasing value utilization, which also reinforces self-likelihood, meaning a reward-controllability positive loop. However, this bias costed an illusion of control. When true controllability is variable and the controller is uncertain, failing to recognize a situation in which one has true controllability harms the outcome for both oneself and for the other person, while wasting inefficient effort when one does not have control only costs one’s own effort. We suspect that this asymmetry makes the fast recognition of controllability more important than the illusion of control, which might justify the presence of the positive bias. We also found that reward increases the controllability signal in the TPJ and striatum to increase value utilization in the vmPFC, which is consistent with our MABC model.

Furthermore, inference bias also played an important role in emotion. A recent study showed that one’s internal control belief increases the amount of happiness and pride after receiving reward [2], and another study showed a similar influence of controllability on guilt-related feelings, such as regret [26]. We reported similar results indicating that multi-agent controllability amplifies the outcome-related mood and that this effect was mediated by striatal activity. These biases overestimate self-controllability after a positive outcome and reduce it after a negative outcome bias one’s mood toward a positive direction. Again, like social learned helplessness we mentioned previously, this suggests how feeling in the social situation is influenced by others’ action-outcome relationship. Studies have suggested that such biases in perceptions of control are characteristics of the normal human brain that are beneficial for mental health [27,28]. Consistently, we have found that people with lower bias are prone to feel more guilt in their daily lives. Although speculative, we suspect that the low positive bias in social life could be related to depression, which is known to exhibit excessive guilt in social life [29].

The relationship between bias and guilt could be related to our study design in which both agents share the outcome. In this scenario, an agent might feel guilty because they inferred that their choice induced loss of another person. However, it is also possible that their guild came from the perception that they induced the loss of themselves. Our study design does not allow us to discriminate these two possibilities. Therefore, this interesting question could be investigated in future studies such that the outcome only goes to one of two agents.

One point that should be noted when interpreting our study is that our design assumed a situation such that only one person can control the situation. However, in the real world, there are other situations in which no one controls the outcome or the cooperation of multiple people, including oneself, influences the outcome, such as in cooperative games in game theory [30]. We expect that extending our model to such a situation could be interesting, and we speculate that in a cooperative situation, there could be multiple levels of controllability, including the overall controllability of the group and relative controllability of the self within the group. Furthermore, one of the limitations of our task is that although we had two sets of tasks having different controllability changing schedules that were randomly assigned across participants, both sets started with a self-controllable block, which might have allowed people to learn a self-controllable state first and an uncontrollable state next. However, this might have resulted in an undesirable blocking effect.

In conclusion, our study advanced previous studies by showing a novel neurocomputational mechanism of controllability inference in the multi-agent setting and its influence on behavior and emotion. The MABC model showed that people utilize both self-and other people’s action-outcome information to compute self-likelihood and other-likelihood by recruiting the frontostriatal regions and right MTG, respectively. Then, multi-agent controllability was inferred by integrating these two likelihood information in the TPJ and striatum. This inferred controllability information was leveraged to increase value utilization in the vmPFC, which resulted in optimal behavior. These results generalize the role of the striatum and vmPFC from single-agent controllability to multi-agent controllability such that involvement of the TPJ in addition to the striatum might help integrate self- and other-related information. We finally showed that innate positive bias facilitated behavioral adaptation in the controllability-changing environment at the cost of illusory control. Furthermore, such bias was related to negative social emotions such as guilt.

In the complex social world with multiple people involved in decision-making situations, identifying our controllability is difficult, and various kinds of psychiatric disorders, such as depression and anxiety, could arise under such complexity. Therefore, by showing how we perceive controllability in the multi-agent setting and how it affects our subsequent behavior and emotion, we believe that our results could benefit research on psychiatric disorders.

## Materials and methods

### Ethics statement

All participants provided written informed consent to participate in the experiment. The study was approved by the university Institutional Review Boards of the KAIST (approval number: KH2017-58) in accordance with the Declaration of Helsinki.

### Experimental design

#### Participants

One hundred and three healthy participants (32 females, mean age of 23.8 ± 2.7 years) without current neurologic or psychiatric disorders from the general university population of Korea Advanced Institute of Science and Technology (KAIST) volunteered and completed the experiment. Among the one hundred and three participants, thirty (8 females, mean age of 24.0 ± 2.7 years) performed the experiment in the MRI scanner.

#### Experimental paradigm: Two-person decision-making task with changing controllability

At the beginning of the experiment, two participants of the same sex met in the experiment room, and an experimenter described the task to both participants. In case one of the two participants was not present for the experiment, one of the experimenters, who was unknown to the participant, participated in the task as a second participant. Participants were informed that they would perform the two-alternative choice task such that each of them would separately make a choice, while the resulting outcome (either reward or loss) would be the only element common to both of them. For example, if they see 0.5 cent as an outcome of some trial (500 won in South Korean money), they will receive 0.25 cent regardless of whether that reward was caused by their own choice (choice of self) or caused by other agent’s (computer) choice. They were told that only one person among them had control over the outcome (person with true controllability; in short, controller), while another person’s choice would not influence the outcome. They were informed that only the controller would have an optimal option that would have a high probability of reward and a suboptimal option that results in loss with high probability, whereas there was no optimal option for a person who was not in control. Therefore, the choice made by the controller was important for both participants, while the choice made by the person without control was irrelevant. Participants also noticed that the person in control was not fixed and sometimes could be switched. Furthermore, participants were told that for the controller, the optimal and suboptimal option could be changed during the task. After describing the experiment, participants were placed in two separate rooms to perform the task. In the fMRI experiment (N = 30), one participant performed the task in the fMRI scanner, while another participant performed the task in a separate room without a fMRI scanner. A total of two task sessions were performed by participants, and each task was composed of 100 trials.

In each trial, both participants were asked to choose between two options that appeared on the upper half of the screen (Fig 1A). The option that participants chose turned red. After participants chose one option, the choice of other person appeared on the lower half of the screen, whose choice was colored blue, and the outcome (reward or loss) appeared on the center of the screen. Although participants were told that the choice that appeared on the screen was the choice made by another participant, the actual choice was made by an artificial reinforcement learning agent that was designed to learn the action-outcome contingency (learning rate = 0.3) and make decisions (inverse temperature = 1.5) through the Rescorla-Wagner rule (see *S1 Text* for rationale behind using interaction with artificial agent rather than using interaction between two real subjects).

Importantly, only one participant (the controller) could choose an option that resulted in a reward with high probability (optimal option, 80% reward, 20% loss), while another option resulted in a loss with high probability (suboptimal option, 20% loss, 80% reward). However, another participant (the person not in control) did not influence the outcome. Therefore, an optimal option was defined only when the participant was a controller. The person in control changed occasionally during the task every 20–30 trials such that there were four controllable blocks and four uncontrollable blocks (artificial agent has a controllability) for each participant. To assess participants’ perceived controllability, they were asked who caused the outcome after receiving outcomes every two or three trials (causality choice, Task 2). Note that an optimal option for the participant existed only in the self-controllable block, and the location of the optimal option can be different in different self-controllable blocks (e.g., left in the first self-controllable block and right in the third self-controllable block; Fig 1B). There was no ‘optimal choice’ for the participant self in the other-controllable block (only optimal choice for the artificial agent exists). In summary, a true controllability change occurred between self-controllable blocks and other controllable blocks, while a change in the optimal option occurred between different self-controllable blocks, as an optimal option existed only within these blocks.

Furthermore, because we were also interested in whether inferred controllability influenced mood, in Task 1, participants were asked to rate their mood every two or three trials instead of asking who caused the outcome. There was a tutorial session before the main task in which participants performed two kinds of tasks (10 trials each) before the main experiment. After finishing the main experiment, participants completed a postexperimental survey including a psychological scale TOSCA-3 (*S1 Text*) [31] to measure participants’ self-conscious affect, especially regarding guilt (mean guilt score: 62.9 ± 6.4).

### Behavior analyses

#### Definition of correlation index

We defined the CIx of each trial using the action-outcome relationship of the two consecutive trials. CIx = 1 at trial k indicates the correlated action-outcome relationship of trials k-1 and k, while CIx = 0 indicates an uncorrelated relationship. CIx was defined for the self (CIx_self_) and for another (CIx_other_) participant. More specifically, we defined trial k as CIx = 0 if the same actions in two consecutive trials resulted in different outcomes (e.g., “left” at trial k-1 resulted in reward and “left” at trial k resulted in loss) or different actions in consecutive trials resulted in the same outcomes (e.g., “left” at trial k-1 resulted in reward and “right” at trial k also resulted in reward). In contrast, CIx = 1 if two of the same consecutive actions resulted in the same outcome or two different actions resulted in different outcomes. Because we instructed participants that if one option was optimal, then the other option was suboptimal, we assumed that even in the latter case (e.g., “left” at trial k-1 resulted in reward and “right” at trial k also resulted in loss), “left” would be consistently associated with reward, while “right” would be consistently associated with loss.

#### Computational model of inferred multi-agent controllability

We derived the MABC model in which an agent updates the inferred controllability at trial k by computing the self- and other’s action-outcome relationship for trial k, which was constrained by the prior (Fig 3A and S1 Table) as follows:

$$\begin{array}{l} p^{\left(k\right)}(z^{\left(k\right)}=1|A_{self}^{\left(k\right)},\;A_{other}^{\left(k\right)},\;O^{\left(k\right)})=\frac{p^{\left(k\right)}(O^{\left(k\right)}|z^{\left(k\right)}=1,\;A_{self}^{\left(k\right)})\hat{p^{\left(k\right)}}(z^{\left(k\right)}=1)}{p^{\left(k\right)}(O^{\left(k\right)}|z^{\left(k\right)}=1,\;A_{self}^{\left(k\right)})\hat{p^{\left(k\right)}}(z^{\left(k\right)}=1)+p^{\left(k\right)}(O^{\left(k\right)}|z^{\left(k\right)}=0,\;A_{other}^{\left(k\right)})\hat{p^{\left(k\right)}}(z^{\left(k\right)}=0)}\\ {}*note\;that\;p^{\left(k\right)}(z^{\left(k\right)}=0|A_{self}^{\left(k\right)},\;A_{other}^{\left(k\right)},\;O^{\left(k\right)})=1-p^{\left(k\right)}(z^{\left(k\right)}=1|A_{self}^{\left(k\right)},\;A_{other}^{\left(k\right)},\;O^{\left(k\right)}) \end{array}$$ (4)

(For the generalized MABC model for the situation including three or more agents, see *S1 Text*)

In this model, latent variable z represents an internally inferred controllability state such that z = 1 if the participant oneself is in control or 0 if the participant’s partner is in control. Thus, p(z = 1) means the probability that the participant (self) has control over the outcome. Note that because we informed participants that one of two people was always in control, sometimes p(z = 1) and p(z = 0) were equal to 1. A_self_ and A_other_ represent the participant’s action and the other person’s (partner) action, respectively, which could be either left or right, and O represents a shared outcome for both participants that could be reward or loss. Therefore, *p*(*z*|*A*_*self*_, *A*_*other*_, *O*) represents the posterior controllability probability of the actions of self and of others and the outcome. $\hat{p}(z=1)$ represents a prior prediction of the controllability. This model shows that an agent’s inferred controllability increases by the ratio between the likelihood of the current outcome given one’s own (current) action (self-likelihood), which is represented as $p^{\left(k\right)}(O^{\left(k\right)}|z^{\left(k\right)}=1,\;A_{self}^{\left(k\right)})$, and the likelihood of the current outcome given another’s action (other-likelihood), which is represented as $p^{\left(k\right)}(O^{\left(k\right)}|z^{\left(k\right)}=0,\;A_{other}^{\left(k\right)})$ and was constrained by the prior $\hat{p}(z=1)$ and $\hat{p}(z=0)$. Note that all hats (^) used in the equation represent a prediction. Participants made causality choices using the posterior equation $p^{\left(k\right)}(z^{\left(k\right)}=1|A_{self}^{\left(k\right)},\;A_{other}^{\left(k\right)},\;O^{\left(k\right)})$ that was applied to the logistic function parameterized by inverse temperature *β*_*con*_, and the posterior was used as a prior for the next trial to compute predicted controllability.

In our winning Bayesian model selection, the multi-agent Bayesian controllability inferences were biased by the outcome valence as follows:

If the outcome of trial k was reward

$$p^{\left(k\right)}(z^{\left(k\right)}=1|A_{self}^{\left(k\right)},\;A_{other}^{\left(k\right)},\;O^{\left(k\right)})=\frac{exp(bias_{pos})p^{\left(k\right)}(O^{\left(k\right)}|z^{\left(k\right)}=1,A_{self}^{\left(k\right)})\hat{p^{\left(k\right)}}(z^{\left(k\right)}=1)}{\exp\left(bias_{pos}\right)p^{\left(k\right)}(O^{\left(k\right)}|z^{\left(k\right)}=1,A_{self}^{\left(k\right)})\hat{p^{\left(k\right)}}(z^{\left(k\right)}=1)+p^{\left(k\right)}(O^{\left(k\right)}|z^{\left(k\right)}=0,A_{other}^{\left(k\right)})\hat{p^{\left(k\right)}}(z^{\left(k\right)}=0)}$$ (5)

If the outcome of trial k was loss

$$p^{\left(k\right)}(z^{\left(k\right)}=1|A_{self}^{\left(k\right)},\;A_{other}^{\left(k\right)},\;O^{\left(k\right)})=\frac{exp(bias_{neg})p^{\left(k\right)}(O^{\left(k\right)}|z^{\left(k\right)}=1,A_{self}^{\left(k\right)})*\hat{p^{\left(k\right)}}(z^{\left(k\right)}=1)}{\exp\left(bias_{neg}\right)p^{\left(k\right)}(O^{\left(k\right)}|z^{\left(k\right)}=1,A_{self}^{\left(k\right)})\hat{p^{\left(k\right)}}(z^{\left(k\right)}=1)+p^{\left(k\right)}(O^{\left(k\right)}|z^{\left(k\right)}=0,A_{other}^{\left(k\right)})\hat{p^{\left(k\right)}}(z^{\left(k\right)}=0)}$$ (6)

Bias parameters (*bias*_*pos*_, *bias*_*neg*_) amplify the relative influence of self-likelihood compared to other-likelihood on inference if these parameters are positive, whereas the relative influence would be reduced if bias parameters are negative. For example, if the bias parameter increased toward infinity, the inferred controllability would move toward 1, making oneself believe that he or she is always a controller, whereas if it decreased toward negative infinity, the inferred controllability would be 0.

Crucially, one can see that the likelihoods of both self and other can be computed from action-outcome contingencies *p*^(*k*)^(*O*^(*k*)^|*z*^(*k*)^, *A*^(*k*)^) of self and other, which was learned by observing the action and following outcome according to the classical reinforcement learning rule called the Rescorla-Wagner rule [10] described below.

The updated equation of learning contingency between own action and outcome is as follows:

$$p^{\left(k\right)}(O^{\left(k\right)}|z^{\left(k\right)}=1,\;A_{self}^{\left(k\right)})={\hat{p}}^{\left(k\right)}(O^{\left(k\right)}|z^{\left(k\right)}=1,\;A_{self}^{\left(k\right)})+a_{self}(O^{\left(k\right)}-{\hat{p}}^{\left(k\right)}(O^{\left(k\right)}|z^{\left(k\right)}=1,\;A_{self}^{\left(k\right)}))$$ (7)

The updated equation of the other’s action-outcome contingency learning is as follows:

$$p^{\left(k\right)}(O^{\left(k\right)}|z^{\left(k\right)}=0,\;A_{other}^{\left(k\right)})={\hat{p}}^{\left(k\right)}(O^{\left(k\right)}|z^{\left(k\right)}=0,\;A_{other}^{\left(k\right)})+a_{other}(O^{\left(k\right)}-{\hat{p}}^{\left(k\right)}(O^{\left(k\right)}|z^{\left(k\right)}=0,\;A_{other}^{\left(k\right)}))$$ (8)

where *a*_*self*_ and *a*_*other*_ represent the learning rate for oneself and another, respectively. These action-outcome contingencies were updated after the outcome presentation, and updated contingencies were used for likelihood computations. Furthermore, an updated action-outcome contingency of the self was used as a value of each action (left or right) in the next trial as follows:

$${V(A)}^{\left(k+1\right)}={\hat{p}}^{\left(k+1\right)}(O^{\left(k+1\right)}|z^{\left(k+1\right)}=1,\;A^{\left(k+1\right)})$$ (9)

Then, logistic functions were applied to the value of two actions to make outcome-related decisions.

In the winning model, predicted controllability influenced behavioral policy during outcome-related decision making. If the predicted controllability was high, the decision policy was changed to utilize more value-related information such that the probability of choosing the high-value option was increased given the same VD, while a low predictive controllability enhanced value-irrelevant random choices. Originally, participants made outcome-related decisions at trial k as follows:

$$p({A_{self}}^{\left(k\right)}=Right)=\frac{1}{1+\exp\left(-\beta (V(A_{self}^{\left(k\right)}=Right)-V(A_{self}^{\left(k\right)}=Left))\right)}\;where\;{V(A)}^{\left(k\right)}={\hat{p}}^{\left(k\right)}(O^{\left(k\right)}|z^{\left(k\right)}=1,\;A^{\left(k\right)})$$ (10)

Usually, the inverse temperature *β* is a constant parameter that does not change trial-by-trial. If *β* = 0, the probability of both actions (right, left) is equal to 0.5, meaning a value-irrelevant random decision. However, if *β* goes to infinity, participants will always choose the action having a higher value (extreme value-based decision making). Therefore, *β* determines the degree of value dependency on the decision. Because we expected that such value dependency would be modulated by the predicted controllability, in the winning model, the predicted controllability influenced *β* of trial k (*β*^(*k*)^) as follows:

$$\beta^{\left(k\right)}=\beta_{0}{\left(\frac{\hat{p^{\left(k\right)}}(z^{\left(k\right)}=1)}{\hat{p^{\left(k\right)}}(z^{\left(k\right)}=0)}\right)}^{\tau }$$ (11)

where *β*_0_ is the baseline inverse temperature and *τ* is the controllability-dependent temperature modulation parameter, which is not an inverse temperature itself but modulates inverse temperature *β*^(*k*)^ according to predicted controllability $\hat{p^{\left(k\right)}}(z^{\left(k\right)}=1)$. If *τ*>0, *β*^(*k*)^ increases according to the ratio between the predictive controllability of the self and others; thus, predictive controllability enhances the value-dependent decision, while if *τ*<0, predictive controllability decreases the value-dependent decision. If *τ* = 0, predictive controllability does not change the inverse temperature from its baseline (*β*_0_); thus, predictive controllability does not influence the degree of value-based decisions (please see S2C Fig for an intuitive explanation regarding a relationship between predictive controllability, *τ* and *β*^(*k*)^).

Additionally, we suspected that participants’ usage of inferred controllability of the previous trial in the controllability prediction of the next trial might not be 100%. For example, one might think that “Although I think I was in control for the previous trial, it could be different in the current trial.”. Therefore, we assumed a “drift” of multi-agent Bayesian controllability inference that approached half such that

$$\hat{p^{\left(k+1\right)}}(z^{\left(k+1\right)})=(1-\theta )p^{\left(k\right)}(z^{\left(k\right)}|A_{self}^{\left(k\right)},\;A_{other}^{\left(k\right)},\;O^{\left(k\right)})+0.5\theta$$ (12)

where *θ* is the drift parameter that links the inference of the controllability at trial k and the prediction of the controllability at trial k+1 such that if some participants’ *θ* is 1, their inferred controllability of the previous trial would not influence the controllability prediction.

To summarize, in the winning model, participants learned of action-outcome contingencies of both self and other by incremental learning, and such action-outcome contingencies were used to calculate self-likelihood and other-likelihood. Both likelihoods were integrated to infer controllability based on Bayesian inference such that the relative likelihood ratio between self and other and the prior controllability determined the posterior inferred controllability. Additionally, biases affecting the inference were considered. Finally, the learned action-outcome contingency was used as value information for the outcome-related choice, and the predicted controllability arbitrated between the value-irrelevant random decision and the value-dependent decision.

#### Model space description

Including the winning model, we constructed thirty-one computational models such that each model represented the corresponding computational hypotheses (S2A Fig). Descriptions regarding other models can be found in the *S1 Text*.

#### Model fitting and comparison

Thirty-one computational models were fit to the participants’ choice patterns in the variational Bayesian scheme using the Translational Algorithms for Psychiatry-Advancing Science (TAPAS) toolbox [32]. Note that our computational models were fitted to jointly explain both outcome-related and causality choices.

First, the prior distributions of each parameter were specified (S1 Table). Then, the maximum a posteriori parameters of each model were obtained by optimizing the log-joint probability function of the prior model and the generative model (each computational model) given the response data using a quasi-Newton optimization algorithm [33]. Then, the negative variational free energy, which was an approximation of the log-model evidence (LME), was calculated using the Laplace approximation [34]. Using this negative variational free energy as the proxy for the LME, models were compared by random-effects Bayesian model selection (RFX-BMS), the procedure of which was implemented using the Variational Bayesian Analysis (VBA) toolbox [35]. This RFX-BMS procedure treats the occurrence of the model within each participant as a multinomial random variable with a Dirichlet conjugate prior [11]. By estimating the parameters of the Dirichlet posterior, we were able to calculate the estimated model frequencies, and the exceedance probabilities (EPs) of one model were more likely than another [36]. Furthermore, by correcting the EPs for the Bayesian omnibus risk, which quantifies the probability that the model frequencies are equal, we obtained the protected EPs (PEPs) of the models. These PEPs were calculated using the equations presented in a previous paper [11]. Note that parameters that were constrained to be located between 0 and 1 were estimated in a logit space (*a*_*self*_, *a*_*other*_, *θ*), and the parameters that were constrained to be positive (β_0_, β_con_) were estimated in log space. Additionally, parameter recovery simulation of the winning model [37] was performed to determine whether the parameters of the model were reliably estimated (*S1 Text* and S2A Fig).

#### Testing correlation between choice optimality and bias parameters

Considering that controllability did not affect value learning but affected the utilization of the value (value-dependent decision making) in our computational model, we expected that such an effect could be more prominent once an agent sufficiently learned about the value. Therefore, we divided the self-controllable block into two halves, and then a correlation between the positive/negative bias parameter and optimal choice proportion in the first/second half was tested separately.

### fMRI analyses

#### MRI acquisition

MRI acquisition was performed using a Siemens Verio Syngo 3T MR scanner (Siemens Healthcare, Erlangen, Germany). A total of 422 functional MRI image volumes per session (2 sessions) were acquired using a T2 * 2D multiband echo-planar imaging (EPI) sequence (repetition time [TR] = 1500 ms, echo time [TE] = 32, flip angle = 50°, voxel size = 2 × 2 × 2 mm^3^, FOV = 225 mm, multiband acceleration factor = 4, and number of slices = 64). A field map was also acquired for distortion correction. During fMRI data acquisition, respiration and pulse data were acquired using a breathing belt and pulse oximetry for all participants but three; for these three participants, data were not acquired due to a technical problem (subject numbers: 3, 23, 30). A high-resolution structural image was obtained using a T1-weighed sequence (TI = 1000 ms, TR = 2400 ms, TE = 2.02 ms, flip angle = 8°, voxel size = 1 × 1 × 1 mm^3^, and FOV = 224 mm).

#### fMRI preprocessing

All fMRI data preprocessing and analyses were performed using Statistical Parametric Mapping 12 (SPM12) [38]. During preprocessing, spatial realignment, unwarping using individual field maps, normalization to Montreal Neurology Institute (MNI) space and smoothing with a 5-mm full-width at half maximum (FWHM) Gaussian kernel were applied to the raw functional images. Additionally, 18 physiological noise regressors were computed using the PhysIO toolbox [39], except three participants who failed to obtain physiological data.

#### General linear model for the model-based fMRI analyses

A first-level general linear model (GLM) in SPM12 [38] was used to model BOLD signals during the task. Our computational model showed that multi-agent controllability was inferred after participants updated their belief regarding the action-outcome relationship, meaning that multi-agent controllability inference might occur after the presentation of the outcome. Therefore, in the first GLM (GLM1), the following events were modeled as stick functions, which were convolved with the canonical hemodynamic response function of the SPM. Those events were 1) one second after the outcome—which was the assumed time of the multi-agent controllability inference (we also tested 0 s after the outcome and 2 s after the outcome in separate GLMs), 2) the time after the outcome presentation (0 s after the outcome)—which was the assumed time of the action-outcome relationship update and 3) the time after the cue presentation. Low-frequency drifts were removed using a high-pass filter (128 s cutoff). All parametric modulators in GLM1 were derived from the winning MABC model in the behavioral analyses section. LCR was defined as a log ratio between the probability that the participant oneself was in control and the probability that another person was in control. If the participant inferred that the controller was a participant, LCR was greater than 0, while LCR<0 had the opposite meaning. The LCR was represented as follows:

$$LCR=log\left(\frac{p^{\left(k\right)}(z^{\left(k\right)}=1)}{p^{\left(k\right)}(z^{\left(k\right)}=0)}\right)$$ (13)

The LCR was used as a parametric modulator one second after the outcome, and the self-reward prediction error (RPE) that occurs during action-outcome contingency learning was used as a parametric modulator at the presentation of the outcome. Note that the RPE of the other person was not used as a parametric modulator because of the severe correlation between self-RPE and other-RPE.

Finally, because we expected a positive interaction between VD-related activity and predicted controllability, we included three parametric modulators at the onset of the cue: the log-predicted controllability ratio (LCR_pred_), VD and the interaction between VD and LCR_pred._ Parametric modulators were not orthogonalized [40]. Twenty-four regressors of no interest, including 6 motion-related regressors and 18 physiological noise regressors (except three participants who failed to obtain physiological data), were also included. Finally, first-level GLM estimates were used as an input for the second-level GLM with a one-sample *t*-test design for the regressors of interest using each participant’s sex as a covariate. Our report of the parametric modulation analysis was based on a cluster-defining threshold (CDT) of p<0.001 [41].

Additionally, because we selected an arbitrary time of one second after the outcome as the time that controllability inference might occur, we suspected that the inference signal might be larger at earlier or later time points. To test this possibility, we performed additional GLMs by setting the timing of the multi-agent controllability inference as 0 or 2 s and compared the mean beta values of the multi-agent controllability inference signal (LCR).

GLM2 used in the Bayesian model selection was modeled similarly to GLM1 except that the LCR and LCR_pred_ were computed from the inferred controllability that was derived from the single-agent controllability model (model 4 in S2A Fig).

Finally, in GLM3, to find regions related to self-likelihood and other-likelihood computation, the event was modeled one second after the outcome, and the parametric modulators of both self-likelihood and other-likelihood derived from the winning MABC model were included at that time.

## Supporting information

S1 Fig **(A) True controllability structure and participants’ causality choice proportion. (B) Other agent (computer)’s learning of optimal option in other-controllable block.** Here, we plotted 1) other agents’ learning of the optimal option in another controllable block (blue line), showing that this agent appropriately learned its optimal option in other-controllable block. Other lines represent belief of self that optimal option in the previous self-block is still an optimal option and inferred controllability which were extracted by winning MABC model.
(TIF)

S2 Fig **(A) Candidate computational models of controllability inference.** Thirty-one computational models of controllability inference were constructed. The winning model in the Bayesian model selection was multi-agent Bayesian controllability with bias and controllability-induced value utilization (Model 12, protected exceedance probability = 1). **(B) Model recovery results.** Nineteen models, including the winning MABC model (model 12, marked as red square), were well recovered (bright yellow color in diagonal element) in the model recovery simulation, while the other 12 models were not properly recovered. **(C) Modulation of the relationship between the predicted controllability and inverse temperature β by parameter τ.** If τ >0, the predicted controllability increased the inverse temperature, which subsequently increased the value-based decision. However, if τ <0 predicted controllability decreased inverse temperature and if τ = 0, predicted controllability did not influence inverse temperature. In this simulation, the baseline inverse temperature β_0_ was set to 1, and the predicted controllability was only tested between 0.1 and 0.9 since parameter θ did not allow the predicted controllability to approach 0 or 1.
(TIF)

S3 Fig Parameter recovery simulation results. Recovered parameters were well correlated with all sampled parameters (all p<0.0001, N = 5000).
(TIF)

S4 Fig Effect of multi-agent controllability on trial-by-trial mood and social emotion. **(A)** We showed that participants’ trial-by-trial mood fluctuation was influenced by the valence of an outcome multiplied by multi-agent controllability. This model explained participants’ real mood trajectory well. **(B)** The controllability-amplified outcome (CMO) parameter was significantly greater than zero (N = 103). **(C)** Participants with a high negative bias parameter (meaning that they tend to think that they had a control for the negative outcome) had a tendency to feel more guilt in their daily life (N = 103). The error bar represents the standard error of the mean (SEM).
(TIF)

S5 Fig Regions involved in other-likelihood computation. The right middle temporal lobe was involved in detecting a decrease in other-likelihood.
(TIF)

S6 Fig Regions involved in self-likelihood computation. The ventromedial prefrontal cortex (right), ventral striatum (middle) and hippocampus (left) were involved in the computation of self-likelihood using one’s own action-outcome relationship.
(TIF)

S7 Fig Granger causality analysis results. Granger causality (GC) analyses between the TPJ and striatum showed that only Granger causality from the TPJ to the striatum was significant, while Granger causality in the reverse direction was not. The histogram represents the distribution of the Granger causality generated from permutation, the red line represents the original Granger causality, and the red star (*) represents the significant Granger causality.
(TIF)

S1 Table Prior and posterior of the winning model parameters. Parameters were transformed into the appropriate estimation space, and then the estimation process was performed. Finally, the estimated parameters were transformed back into the original space.
(DOCX)

S2 Table Results of fMRI analyses (GLM1). (DOCX)

S3 Table Results of fMRI analyses (GLM3). (DOCX)

S1 Text Supplementary methods and results. (DOCX)
