# Supplementary figures and images for: Neurocomputational mechanism of controllability inference under a multi-agent setting
Source: PLoS Comput Biol. 2021 Nov 9;17(11):e1009549. doi: 10.1371/journal.pcbi.1009549 (PMC8604335; doi:10.1371/journal.pcbi.1009549)

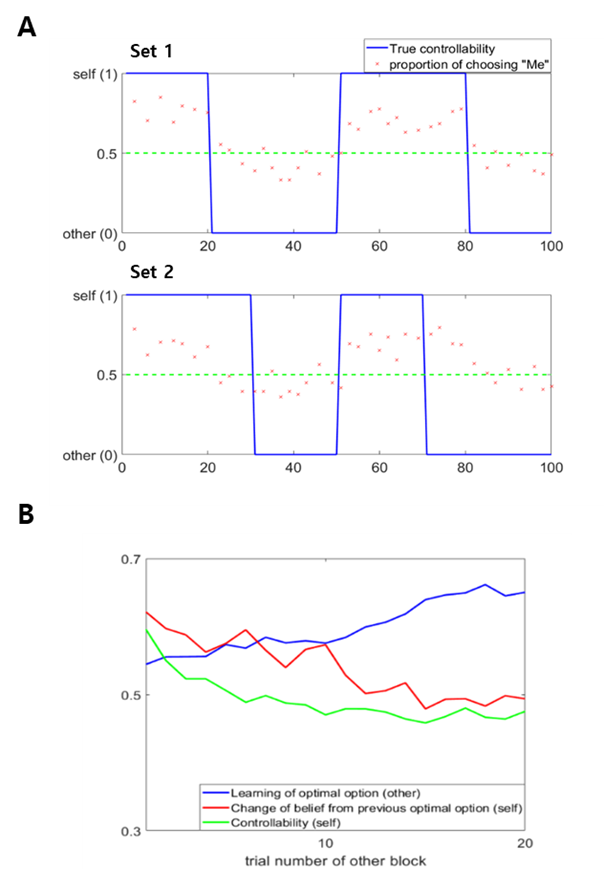

Supplement: S1 Fig — (A) True controllability structure and participants’ causality choice proportion. (B) Other agent (computer)’s learning of optimal option in other-controllable block. Here, we plotted 1) other agents’ learning of the optimal option in another controllable block (blue line), showing that this agent appropriately learned its optimal option in other-controllable block. Other lines represent belief of self that optimal option in the previous self-block is still an optimal option and inferred controllability which were extracted by winning MABC model. (TIF) [file pcbi.1009549.s001.tif]

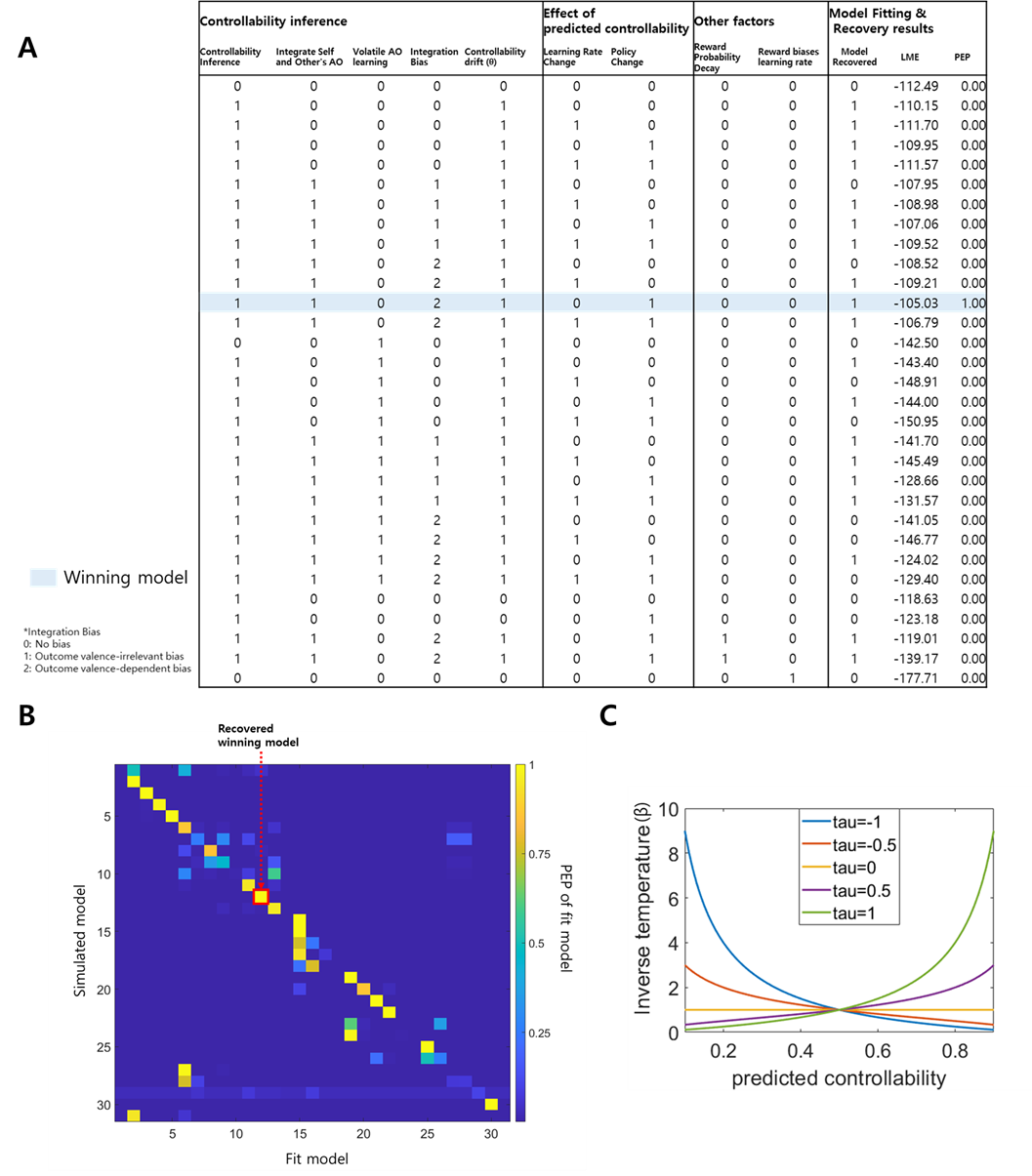

Supplement: S2 Fig — (A) Candidate computational models of controllability inference. Thirty-one computational models of controllability inference were constructed. The winning model in the Bayesian model selection was multi-agent Bayesian controllability with bias and controllability-induced value utilization (Model 12, protected exceedance probability = 1). (B) Model recovery results. Nineteen models, including the winning MABC model (model 12, marked as red square), were well recovered (bright yellow color in diagonal element) in the model recovery simulation, while the other 12 models were not properly recovered. (C) Modulation of the relationship between the predicted controllability and inverse temperature β by parameter τ. If τ >0, the predicted controllability increased the inverse temperature, which subsequently increased the value-based decision. However, if τ <0 predicted controllability decreased inverse temperature and if τ = 0, predicted controllability did not influence inverse temperature. In this simulation, the baseline inverse temperature β0 was set to 1, and the predicted controllability was only tested between 0.1 and 0.9 since parameter θ did not allow the predicted controllability to approach 0 or 1. (TIF) [file pcbi.1009549.s002.tif]

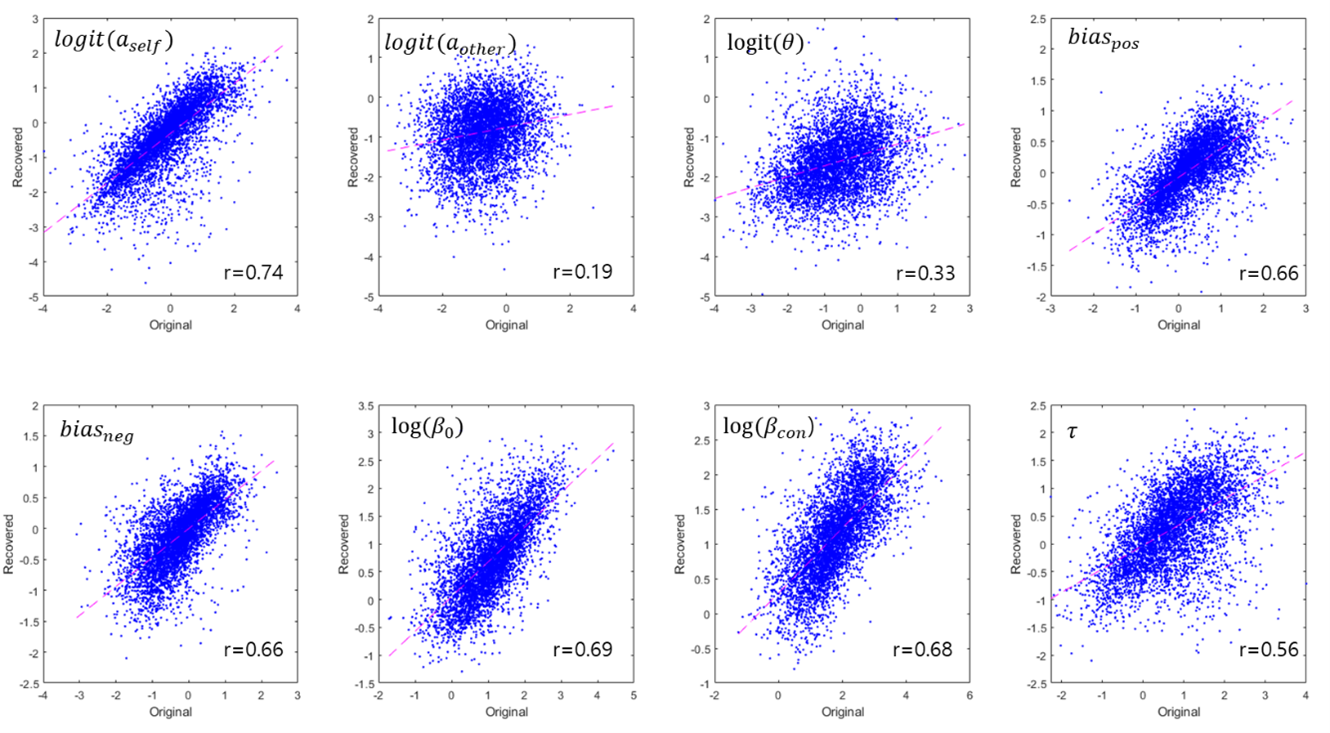

Supplement: S3 Fig — Recovered parameters were well correlated with all sampled parameters (all p<0.0001, N = 5000). (TIF) [file pcbi.1009549.s003.tif]

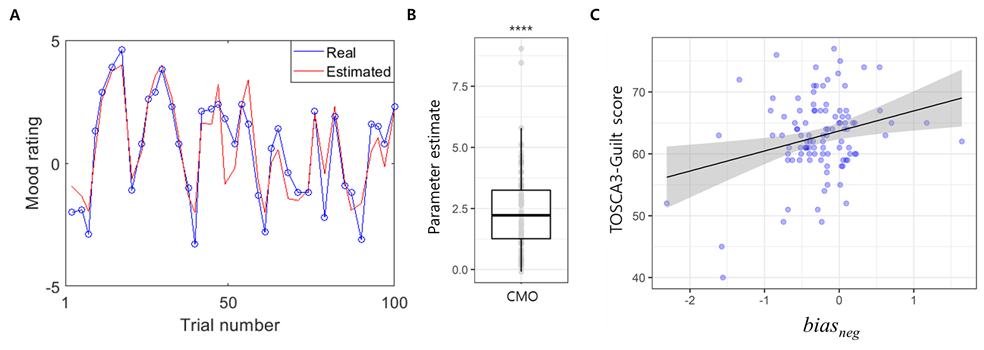

Supplement: S4 Fig — (A) We showed that participants’ trial-by-trial mood fluctuation was influenced by the valence of an outcome multiplied by multi-agent controllability. This model explained participants’ real mood trajectory well. (B) The controllability-amplified outcome (CMO) parameter was significantly greater than zero (N = 103). (C) Participants with a high negative bias parameter (meaning that they tend to think that they had a control for the negative outcome) had a tendency to feel more guilt in their daily life (N = 103). The error bar represents the standard error of the mean (SEM). (TIF) [file pcbi.1009549.s004.tif]

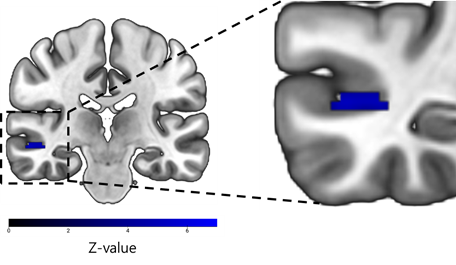

Supplement: S5 Fig — The right middle temporal lobe was involved in detecting a decrease in other-likelihood. (TIF) [file pcbi.1009549.s005.tif]

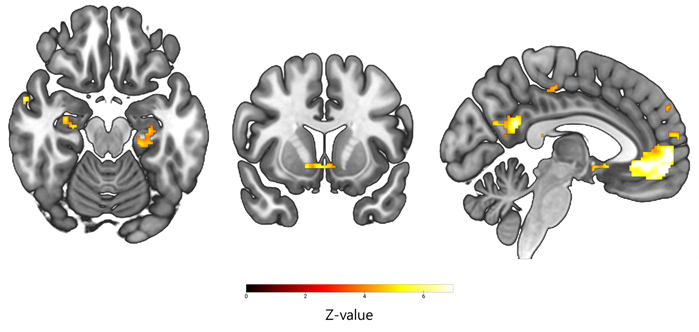

Supplement: S6 Fig — The ventromedial prefrontal cortex (right), ventral striatum (middle) and hippocampus (left) were involved in the computation of self-likelihood using one’s own action-outcome relationship. (TIF) [file pcbi.1009549.s006.tif]

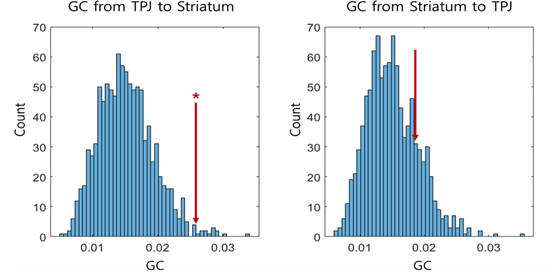

Supplement: S7 Fig — Granger causality (GC) analyses between the TPJ and striatum showed that only Granger causality from the TPJ to the striatum was significant, while Granger causality in the reverse direction was not. The histogram represents the distribution of the Granger causality generated from permutation, the red line represents the original Granger causality, and the red star (*) represents the significant Granger causality. (TIF) [file pcbi.1009549.s007.tif]
