# Supplementary material for: Neurocomputational mechanism of controllability inference under a multi-agent setting
Source: PLoS Comput Biol. 2021 Nov 9;17(11):e1009549. doi: 10.1371/journal.pcbi.1009549 (PMC8604335; doi:10.1371/journal.pcbi.1009549)
Supplement: S1 Table — Parameters were transformed into the appropriate estimation space, and then the estimation process was performed. Finally, the estimated parameters were transformed back into the original space. (DOCX) [file pcbi.1009549.s008.docx]

**S1 Table. Prior and posterior of the winning model parameters**

| **Parameter**  **(original)** | **Parameter**  **(estimation space)** | **Description** | **Possible range of original parameter** | **Prior mean**  **(estimation space)** | **Prior variance**  **(estimation space)** | **Posterior mean**  **(estimation space)** | **Posterior variance**  **(estimation space)** |
| --- | --- | --- | --- | --- | --- | --- | --- |
| $\boldsymbol{a}_{\boldsymbol{self}}$ | $\boldsymbol{logit(}\boldsymbol{a}_{\boldsymbol{self}}\boldsymbol{)}$ | Action-outcome learning rate of self | [0, 1] | -1 | 2 | -0.16 | 1.00 |
| $\boldsymbol{a}_{\boldsymbol{other}}$ | $\boldsymbol{logit(a}_{\boldsymbol{other}}\boldsymbol{)}$ | Action-outcome learning rate of other’s | [0, 1] | -1 | 2 | -0.71 | 0.61 |
| $\boldsymbol{\theta}$ | $\boldsymbol{logit(\theta}$**)** | Drift parameter | [0, 1] | -1 | 3 | -0.65 | 0.78 |
| $\boldsymbol{bia}\boldsymbol{s}_{\boldsymbol{pos}}$ | $\boldsymbol{bia}\boldsymbol{s}_{\boldsymbol{pos}}$ | Biased integration of likelihoods after reward | [-∞, +∞] | 0 | 1 | 0.23 | 0.26 |
| $\boldsymbol{bia}\boldsymbol{s}_{\boldsymbol{neg}}$ | $\boldsymbol{bia}\boldsymbol{s}_{\boldsymbol{neg}}$ | Biased integration of likelihoods after loss | [-∞, +∞] | 0 | 1 | -0.26 | 0.27 |
| $\boldsymbol{\beta}_{\mathbf{0}}$ | $\mathbf{log(}\boldsymbol{\beta}_{\mathbf{0}}\mathbf{)}$ | Baseline inverse temperature of outcome-related decision | [0, +∞] | log 3 | 1 | 1.01 | 0.37 |
| $\boldsymbol{\beta}_{\boldsymbol{con}}$ | ${\mathbf{log(}\boldsymbol{\beta}}_{\boldsymbol{con}}\boldsymbol{)}$ | Inverse temperature of causality choice | [0, +∞] | log 3 | 1 | 1.79 | 0.70 |
| $\boldsymbol{\tau}$ | $\boldsymbol{\tau}$ | Value-dependency modulation parameter | [-∞, +∞] | 0 | 1 | 0.74 | 0.75 |

* Parameters were transformed into the appropriate estimation space, and then the estimation process was performed. Finally, the estimated parameters were transformed back into the original space.
