# Supplementary material for: Neurocomputational mechanism of controllability inference under a multi-agent setting
Source: PLoS Comput Biol. 2021 Nov 9;17(11):e1009549. doi: 10.1371/journal.pcbi.1009549 (PMC8604335; doi:10.1371/journal.pcbi.1009549)
Supplement: S2 Table — (DOCX) [file pcbi.1009549.s009.docx]

**S2 Table.** **Results of fMRI analyses (GLM1).**

| **Regions activated by inferred multiagent controllability** | | | | | | | | | | | | | |  |
| --- | --- | --- | --- | --- | --- | --- | --- | --- | --- | --- | --- | --- | --- | --- |
| **Cluster** | | **Cluster**  **p-value**  **(FWE corrected)** | | | **No. of voxels** | | | **MNI**  **coordinates**  **(x, y, z)** | | | **Voxel**  **Z-Value** | | |  |
| **Right striatum** | | <0.001 | | | 189 | | | 18,8, -6 | | | 4.82 | | |  |
|  |  |  |  |  |  |  |  | 20, 8, -10 | | | 4.77 | | |  |
|  |  |  |  |  |  |  |  | 26, 28, 4 | | | 3.82 | | |  |
| **Left TPJ** | | 0.032 | | | 88 | | | -40, -22, 26 | | | 4.37 | | |  |
|  |  |  |  |  |  |  |  | -52, -18, 34 | | | 3.75 | | |  |
|  |  |  |  |  |  |  |  | -58, -14, 30 | | | 3.67 | | |  |
| **Regions activated by value differences** | | | | | | | | | | | | | |  |
| **Cluster** | | **Cluster**  **p-value**  **(FWE corrected)** | | | **No. of voxels** | | | **MNI**  **coordinates**  **(x, y, z)** | | | **Voxel**  **Z-Value** | | |  |
| **vmPFC** | | <0.001 | | | 745 | | | 0, 52, -12 | | | 5.18 | | |  |
|  |  |  |  |  |  |  |  | -2, 58, 4 | | | 5.17 | | |  |
|  |  |  |  |  |  |  |  | -4, 44, -16 | | | 4.81 | | |  |
| **Left superior temporal gyrus** | | 0.049 | | | 85 | | | -54, 0, 10 | | | 4.79 | | |  |
|  |  |  |  |  |  |  |  | -58, -10, 8 | | | 4.02 | | |  |
|  |  |  |  |  |  |  |  | -58, -4, 2 | | | 3.47 | | |  |
| **Left precentral gyrus** | | <0.001 | | | 423 | | | -42, -8, 58 | | | 4.58 | | |  |
|  |  |  |  |  |  |  |  | 4, -20, 58 | | | 4.39 | | |  |
|  |  |  |  |  |  |  |  | -32, -20, 70 | | | 4.25 | | |  |
| **Precuneus** | | 0.016 | | | 109 | | | -10, -52, 20 | | | 4.29 | | |  |
|  |  |  |  |  |  |  |  | -4, -52, 14 | | | 4.11 | | |  |
|  |  |  |  |  |  |  |  | -2, -50, 28 | | | 3.99 | | |  |
| **Regions showing VD * predicted controllability interaction** | | | | | | | | | | | | | | |
| **Cluster** | | | **Cluster**  **p-value**  **(Small volume corrected)** | | | **No. of voxels** | | | **MNI**  **coordinates**  **(x, y, z)** | | | **Voxel**  **Z-Value** | | |
| **vmPFC** | | | 0.012 | | | 31 | | | -2, 34, -14 | | | 4.01 | | |
|  |  |  |  |  |  |  |  |  | 0, 32, -10 | | | 3.63 | | |
| **Regions activated by reward prediction error of the self action** | | | | | | | | | | | | |  |  |
| **Cluster** | **Cluster**  **p-value**  **(FWE corrected)** | | | **No. of voxels** | | | **MNI**  **coordinates**  **(x, y, z)** | | | **Voxel**  **Z-Value** | | |  |  |
| **vmPFC** | <0.001 | | | 5303 | | | -8, 50, 6 | | | 7.35 | | |  |  |
|  |  |  |  |  |  |  | -6, 42, -2 | | | 6.02 | | |  |  |
|  |  |  |  |  |  |  | 12, 48, 48 | | | 5.74 | | |  |  |
| **Middle cingulate gyrus** | <0.001 | | | 1827 | | | 0, -28, 42 | | | 4.76 | | |  |  |
|  |  |  |  |  |  |  | -4, -62, 26 | | | 4.74 | | |  |  |
|  |  |  |  |  |  |  | -2, -4, 36 | | | 4.68 | | |  |  |
| **Right superior temporal gyrus** | <0.001 | | | 839 | | | 62, -20, 10 | | | 4.75 | | |  |  |
|  |  |  |  |  |  |  | 42, -30, 20 | | | 4.56 | | |  |  |
|  |  |  |  |  |  |  | 30, -18, 4 | | | 4.41 | | |  |  |
| **Right superior temporal gyrus** | 0.002 | | | 150 | | | 54, 0, 0 | | | 4.69 | | |  |  |
|  |  |  |  |  |  |  | 48, -10, 6 | | | 3.54 | | |  |  |
| **Left angular gyrus** | <0.001 | | | 319 | | | -48, -66, 34 | | | 4.62 | | |  |  |
|  |  |  |  |  |  |  | -42, -72, 38 | | | 4.01 | | |  |  |
|  |  |  |  |  |  |  | -54, -68, 26 | | | 3.90 | | |  |  |
| **Left lateral orbitofrontal cortex** | <0.001 | | | 192 | | | -40, 24, -14 | | | 4.49 | | |  |  |
|  |  |  |  |  |  |  | -26, 28, -14 | | | 4.45 | | |  |  |
|  |  |  |  |  |  |  | -42, 36, -12 | | | 3.96 | | |  |  |
| **Left superior temporal gyrus** | 0.003 | | | 142 | | | -48, -8, -2 | | | 4.34 | | |  |  |
|  |  |  |  |  |  |  | -50, 0, -8 | | | 4.21 | | |  |  |
|  |  |  |  |  |  |  | -56, 4, -16 | | | 3.94 | | |  |  |
| **Right middle temporal gyrus** | 0.017 | | | 106 | | | 54, -4, -28 | | | 4.31 | | |  |  |
|  |  |  |  |  |  |  | 60, 0, -20 | | | 3.95 | | |  |  |
| **Left middle temporal gyrus** | 0.001 | | | 168 | | | -60, -40, -8 | | | 4.22 | | |  |  |
|  |  |  |  |  |  |  | -66, -26, -6 | | | 4.00 | | |  |  |
|  |  |  |  |  |  |  | -66, -18, -4 | | | 3.51 | | |  |  |
| **Left cuneus** | <0.001 | | | 226 | | | -8, -90, 22 | | | 4.21 | | |  |  |
|  |  |  |  |  |  |  | -6, -88, 12 | | | 3.96 | | |  |  |
|  |  |  |  |  |  |  | -16, -94, 20 | | | 3.65 | | |  |  |
| **Left insula** | 0.017 | | | 106 | | | -36, 2, 2 | | | 4.15 | | |  |  |
|  |  |  |  |  |  |  | -42, 6, -4 | | | 3.31 | | |  |  |
|  |  |  |  |  |  |  | -30, 6, 12 | | | 3.30 | | |  |  |
