# Supplementary material for: Neurocomputational mechanism of controllability inference under a multi-agent setting
Source: PLoS Comput Biol. 2021 Nov 9;17(11):e1009549. doi: 10.1371/journal.pcbi.1009549 (PMC8604335; doi:10.1371/journal.pcbi.1009549)
Supplement: S3 Table — (DOCX) [file pcbi.1009549.s010.docx]

**S3 Table. Results of fMRI analyses (GLM3).**

| **Regions activated by decreasing other-likelihood** | | | | | | | | | |
| --- | --- | --- | --- | --- | --- | --- | --- | --- | --- |
| **Cluster** | | **Cluster**  **p-value**  **(FWE corrected)** | | **No. of voxels** | | **MNI**  **coordinates**  **(x, y, z)** | | **Voxel**  **Z-Value** | |
| **Right MTL** | | 0.035 | | 96 | | 56, 22, 8 | | 4.52 | |
|  |  |  |  |  |  | 46, 24, 8 | | 4.32 | |
| **Regions activated by self-likelihood** | | | | | | | | | |
| **Cluster** | | **Cluster**  **p-value**  **(FWE corrected)** | | **No. of voxels** | | **MNI**  **coordinates**  **(x, y, z)** | | **Voxel**  **Z-Value** | |
| **Right vmPFC** | | <0.001 | | 2041 | | -2,40, 18 | | 6.16 | |
| **Posterior cingulate cortex** | | <0.001 | | 616 | | -4, 52,24 | | 5.36 | |
| **Ventral striatum** | | <0.001 | | 264 | | 12,4, 10 | | 5.32 | |
| **Rolandic operculum** | | <0.001 | | 1601 | | 40, 12,20 | | 5.21 | |
| **Right hippocampus** | 0.004 | | 165 | | 30, 12, 16 | | 5.09 | |  |
| **Left superior frontal gyrus** | <0.001 | | 395 | | -14,46,50 | | 5.05 | |  |
| **Right middle temporal gyrus** | <0.001 | | \| 227 \| \| --- \| | | 60,2, 20 | | 4.93 | |  |
| **Left superior temporal pole** | <0.001 | | 259 | | -48,8, 24 | | 4.93 | |  |
| **Middle cingulate cortex** | 0.003 | | 1832 | | 16, 30,26 | | 4.74 | |  |
| **Right superior frontal gyrus** | 0.017 | | 126 | | 14,48,40 | | 4.31 | |  |
| **Right temporal pole** | 0.001 | | 179 | | 38,14, 30 | | 4.22 | |  |
| **Right precuneus** | 0.029 | | 113 | | 16, 30,26 | | 4.74 | |  |
| **Left hippocampus** | <0.001 | | 292 | | -28, 20, 14 | | 4.74 | |  |
| **Left insula** | 0.032 | | 111 | | -40, 12, 2 | | 4.74 | |  |
