# Supplementary material for: Neurocomputational mechanism of controllability inference under a multi-agent setting
Source: PLoS Comput Biol. 2021 Nov 9;17(11):e1009549. doi: 10.1371/journal.pcbi.1009549 (PMC8604335; doi:10.1371/journal.pcbi.1009549)
Supplement: S1 Text — (DOCX) [file pcbi.1009549.s011.docx]

**S1 Text**

**Rationale behind using interaction with artificial agent rather than using interaction between two real subjects**

We used interaction with a standardized artificial agent because we wanted to obtain participants’ responses within a more controlled situation. We expected that an interaction between participants would have more unwanted noise in responses than an interaction between one participant and an artificial agent.

**Description of the TOSCA scale**

We asked participants to complete the TOSCA-3 scale (TOSCA-3; Tangney *et al*., 2000) to evaluate their proneness to guilt in daily life. This questionnaire consists of sixteen brief scenarios that participants would be likely to encounter in day-to-day life. For each scenario, there are a number of associated statements regarding shame and guilt in that situation, and participants have to rate their response to each of those statements using a 5-point scale. Among the five subscores of the TOSCA-3 (shame, guilt, externalization, unconcern and pride), we used the TOSCA guilt score to evaluate guilt (mean (sd)=62.88 ± 6.37).

**Derivation of multi-agent Bayeisan controllability based on Bayesian inference when the number of agents is two (self and one other person)**

The posterior probability that the self is in control $p\left( z=1 | A_{\mathrm{self}},A_{\mathrm{other}},O \right)$given the action of self ($A_{\mathrm{self}})$, the action of other ($A_{\mathrm{other}})$, the common outcome ($O$) and the prior ($p\left( z=1 \right)$) of current trial was derived as follows:

$$p\left( z=1 | A_{\mathrm{self}},A_{\mathrm{other}},O \right)$$

$$=\frac{p\left( O | z=1,A_{\mathrm{self}},A_{\mathrm{other}} \right)p\left( z=1,A_{\mathrm{self}},A_{\mathrm{other}} \right)}{p\left( O | z=1,A_{\mathrm{self}},A_{\mathrm{other}} \right)p\left( z=1,A_{\mathrm{self}},A_{\mathrm{other}} \right)+p\left( O | z=0,A_{\mathrm{self}},A_{\mathrm{other}} \right)p\left( z=0,A_{\mathrm{self}},A_{\mathrm{other}} \right)}$$

$$=\frac{p\left( O | z=1,A_{\mathrm{self}},A_{\mathrm{other}} \right)p\left( z=1,A_{\mathrm{self}},A_{\mathrm{other}} \right)}{p\left( O | z=1,A_{\mathrm{self}} \right)p\left( z=1,A_{\mathrm{self}},A_{\mathrm{other}} \right)+p\left( O | z=0,A_{\mathrm{other}} \right)p\left( z=0,A_{\mathrm{self}},A_{\mathrm{other}} \right)}$$

$$=\frac{p\left( O | z=1,A_{\mathrm{self}},A_{\mathrm{other}} \right)p\left( z=1 \right)p\left( A_{\mathrm{self}},A_{\mathrm{other}} \right)}{p\left( O | z=1,A_{\mathrm{self}} \right)p\left( z=1 \right)p\left( A_{\mathrm{self}},A_{\mathrm{other}} \right)+p\left( O | z=0,A_{\mathrm{other}} \right)p\left( z=0 \right)p\left( A_{\mathrm{self}},A_{\mathrm{other}} \right)} (eq s1)$$

$$=\frac{p\left( O | z=1,A_{\mathrm{self}} \right)p\left( z=1 \right)}{p\left( O | z=1,A_{\mathrm{self}} \right)p\left( z=1 \right)+p\left( O | z=0,A_{\mathrm{other}} \right)p\left( z=0 \right)}$$

$$*p\left( z=1 \right) is independent with p\left( A_{\mathrm{self}},A_{\mathrm{other}} \right) before observing the outcome$$

where $p\left( O | z=1,A_{\mathrm{self}} \right)$ is the self-likelihood and $p\left( O | z=0,A_{\mathrm{other}} \right)$ is the other-likelihood.

**Generalization of the MABC model to the situation with more than three agents (self and two or more other people)**

Although we considered situations in which there is myself and only one other agent, we can apply the MABC model to situations with three or more agents. In this generalized MABC model, other-likelihood $p\left( O | z=0,A_{\mathrm{other}} \right)$ means the probability that any other person except me has caused the current outcome, and z = 0 means that anyone except me has controllability.

Assume that there are N other agents except me and z_o_=k (k $\in$ {1,2,…N}) means that the controller is k th other agent and A_other(k)_ means the action of k th other agent.

Then, other likelihoods in this situation can be represented as follows:

$$p\left( O | z=1,A_{\mathrm{other}} \right)=p\left( \bigcup_{k=1}^{N} (O|z_{o}=k,A_{\mathrm{other}\left( k \right)}) \right)$$

$$=1-p\left( \bigcap_{k=1}^{N} \left( O | z_{o}=k,A_{\mathrm{other}\left( k \right)} \right)^{∁} \right)$$

$$=1- \prod_{k=1}^{N} (1-p\left( O | z_{o}=k,A_{\mathrm{other}\left( k \right)} \right)) (eq s2)$$

where each $p\left( O | z_{o}=k,A_{\mathrm{other}\left( k \right)} \right)$ can be learned from action-outcome observation of k th other agent according to the Rescorla-Wagner rule or other learning rule. Then, this other-likelihood equation can be applied to the original MABC model ($eq s1)$

Note that if k=1, meaning that the number of agents is 1, this becomes the MABC model used in our experiment.

**Model space description**

Here, we describe how thirty-one computational models of controllability inference were constructed by combining six factors: 1) whether people infer controllability; 2) whether people integrate the self- and other-action-outcome relationship to infer controllability or whether their inference solely depends on their own action-outcome relationship; 3) whether the controllability inference was biased according to the outcome valence and 4) whether inferred controllability influenced behavior policy (value-dependent deliberate choice vs. random choice); 5) the possibility that controllability might influence the learning rate of the action-outcome relationship—for example, we expected that increased controllability might increase attention to the choice and outcome to increase the learning rate [1]; and 6) the expectation that action-outcome relationship learning could occur with a consistent learning rate or could occur with a varying learning rate that is increases by the volatility estimate of the probabilistic environment [2].

1) In the model in which an agent does not infer controllability, the causality choice was modeled to be random (0.5 for each action).

2) The model in which an agent infers controllability solely based on his own action-outcome relationship followed the previous study showing that perceived controllability is low if an action randomly produces many outcomes with similar probability while perceived controllability is high [3]. Therefore, we defined controllability here as the absolute difference between the reward probability of two actions as follows:

$$p^{\left( k \right)}\left( z^{\left( k \right)}=1 \right)=$$

$$\left| p^{\left( k \right)}\left( O^{\left( k \right)}=Reward \right|z^{\left( k \right)}=1, A_{self}^{\left( k \right)}=Right \right)-p^{\left( k \right)}\left( O^{\left( k \right)}=Reward \right|z^{\left( k \right)}=1, {A^{'}}_{self}^{\left( k \right)}=Left)|$$

For example, if an agent expects that both right and left actions are equally likely to result in reward (0.5, 0.5), the inferred controllability is zero; however, if an agent expects that the reward probability of one option (right) dominates the other (left) (reward probability=1 and 0 for right and left action), an agent perceives full controllability over the outcome; thus, the inferred controllability is 1.

3) The bias of integrating self-likelihood and other-likelihood on the multi-agent controllability inference (integration bias) could be absent (0 in Fig. S2A), outcome valence-dependent (2 in Fig. S2A) or constant and does not depend on the outcome valence (1 in Fig. S2A).

4) In models in which predicted controllability does not influence behavior policy, the inverse temperature of the outcome-related decision ($\beta_{0}$) was a constant parameter.

5) We suspected that predicted controllability might influence the learning rate of action-outcome contingencies by increasing attention as follows:

$${a_{self}}^{\left( k \right)}=a_{self}\hat{p^{\left( k \right)}}\left( z^{\left( k \right)}=1 \right)$$

$${a_{other}}^{\left( k \right)}=a_{other}\hat{p^{\left( k \right)}}\left( z^{\left( k \right)}=1 \right)$$

where ${a_{self}, a}_{other}$ is the baseline learning rate.

6) Finally, in our task, the “good” and “bad” options for the controller changed across the blocks of trials, reflecting a volatile environment. Therefore, we suspected that participants’ action-outcome contingency learning might be better explained by learning models under volatility. Therefore, in models assuming volatile action-outcome contingency learning of the self, a three-level hierarchical Gaussian filter model (HGF3) [2] was applied, which is one of the state-of-art models of learning under volatility. Briefly, in this model, an agent infers the hierarchical structure of the (possibly) volatile environment that generates the current sensory input [2]. A detailed description of the model can be found in Mathys *et al*., 2014 [2].

Additionally, we tested three types of models (models 27–31 in Fig. S2A): 1) a model in which the agent does not consider changing controllability but only considers the environment to have changing reward probability (model 31). In particular, this model assumes an agent whose learning rate is biased by the reward (rather than biasing controllability inference), 2) models do not have parameter θ (model 27, 28) and 3) models that reward probability drifts to 0.5 by parameter $\theta_{rew}$which is similar to a drift of controllability in our winning MABC model (by parameter θ; model 29, 30).

**Parameter and model recovery simulation**

We performed parameter recovery of the winning model by following the method recommended in a previous paper [4], which suggested parameter recovery using the posterior parameter range. We sampled 5000 combinations of parameters sampled from the posterior distribution of the winning model parameters (normal distribution with posterior mean and variance of the parameters) and compared these parameters with recovered parameters. Recovered parameters were well correlated with all sampled parameters (all p<0.0001, Fig. S3).

Furthermore, we also performed a model recovery analysis of all 31 models we tested. We sampled the model parameter from the posterior parameter distribution 50 times and generated the corresponding 50 responses. Then, these simulated responses were fitted to all 31 computational models, and LME for the corresponding models was acquired. Using this LME, we performed Bayesian model selection. Our winning model was successfully recovered with a PEP of 1. However, among 31 models, 12 models were not recovered appropriately (Fig. S2B).

**Comparing model fit of the task 1 and task 2**

Because task 1 and task 2 involve different rating tasks (mood and causality choice), participants’ explicit tracking of controllability might be different between these two tasks, which might have influenced both the inference and the learning process. If different ratings affected people’s controllability tracking, the model fit of the winning MABC model for task 2, where people have to explicitly track controllability by causality rating, should be better than the model fit for task 1. To test this hypothesis, we compared log model evidence (LME) of this model on choice behavior of task 1 and task 2 (causality choices were not included in this model evidence comparison, since these choices are only included in task 2). The results showed no evidence of a model fit difference between the two tasks (mean LME: task 1 = -38.1, task 2 = -36.9; t[102.00]=1.09, p=0.2775, CIs: -3.54 to 1,03, d=0.854, paired t-test).

**Differential influence of positive bias and negative bias on controllability perception**

We showed that positive bias is related to an illusion of control. Therefore, it is possible that negative bias might reduce controllability in self-controllable blocks (“illusion of losing control”). However, the correlation between _bias_ and the proportion of selecting “Other” in the causality choice in the self-controllable block was insignificant (Pearson r_partial_=-0.08; p=0.4523). Furthermore, _bias_ was not correlated with better perception of other people’s controllability (Pearson r_partial_=-0.06; p=0.5214), which was true when we divided other-controllable blocks into two blocks and tested correlation separately (all p>0.7 in both first and second other-controllable blocks). However, bias_pos_ was correlated with worse performance of perceiving other people’s controllability in both the first and second other-controllable blocks, meaning an illusion of control (Pearson rpartial=-0.30; p=0.0024 and rpartial=-0.28; p=0.0039 for the first and second other-controllable blocks). These results might suggest that the influence of positive bias on controllability perception might be much greater than the influence of negative bias, although the magnitudes of both parameters were not significantly different (t[102]=0.71, p=0.4822 in paired t-test). One likely reason is that most participants experienced reward more frequently than loss (average reward: 59.4% of all trials vs. average loss: 40.3% of all trials; t[102]=22.71, p<0.0001 in paired t-test) because both participants and computer agent were able to learn to choose the optimal option in controllable blocks, which provides more opportunity that controllability inference can be biased by reward.

**Computational model of trial-by-trial mood**

In addition to an effect of positive bias on behavioral adaptability in a volatile controllability environment, we expected that the biases would have a beneficial effect on mental health by 1) decreasing negative feelings that arise due to loss by decreasing perceived controllability and 2) increasing positive feelings that arise due to reward by increasing perceived controllability. Indeed, previous studies showed that when perceived controllability is high, people tend to feel more negative after a bad outcome and more positive after a good outcome compared to the feelings experienced in a low perceived controllability condition [5, 6]. Therefore, we expected that participants’ inferred controllability would amplify the feeling induced by the outcome. To test this hypothesis, we first modeled trial-by-trial mood ratings after the outcome using models that are similar to previous work that modeled moment-to-moment happiness and tested model coefficients to identify each variable’s effect on mood [7]. The optimal model in that study explained the trial-by-trial happiness rating mood fluctuation by a combination of the RPE, expected value (EV) and outcome (O), which assumed an exponential decay of variables [7] as follows:

$$\mathrm{Mood}\left( t \right)=w_{0}+w_{1}\sum_{j=1}^{t} \gamma^{t-j}O_{j}+w_{2}\sum_{j=1}^{t} \gamma^{t-j}{EV}_{j}+w_{3}\sum_{j=1}^{t} \gamma^{t-j}\mathrm{RPE}_{j}$$

where $w_{1}, w_{2}, w_{3}$ are the weights, $w_{0}$ is the intercept representing the baseline mood and γ is the discounting factor.

Based on this model, we developed several other models considering inferred controllability. Because we expected that the feeling induced by the given feedback would be proportional to inferred controllability, we defined a variable called the controllability-multiplied outcome (CMO), which was the outcome (+1 for reward and -1 for loss) multiplied by the inferred controllability of that trial. We expected that the combination of the RPE, EV and CMO would explain participants’ mood better than the previous model in our task.

Additionally, we suspected that the probability of the current outcome given one’s choice (self-likelihood; SL) might influence mood or that SL might amplify outcome-induced mood (SL-multiplied outcome; SLMO). Therefore, we tested twelve models, each having combinations of these six variables as factors as follows:

1. RPE+EV+O (the previous optimal model)
2. RPE+EV+CMO
3. RPE+EV+CMO+O
4. CMO only
5. RPE+EV+O+SL
6. RPE+EV+CMO+SL
7. RPE+EV+CMO+O+SL
8. CMO+SL
9. RPE+EV+SLMO
10. RPE+EV+ SLMO
11. RPE+EV+ SLMO +O
12. SLMO only

Each variable was derived from the winning multi-agent controllability inference model. These models were fitted to each individual by nonlinear least squares using the optimization toolbox in MATLAB (MathWorks, Inc.) and compared by RFX-BMS [8].

We showed that participants’ trial-by-trial mood fluctuation was influenced by the valence of an outcome multiplied by the multi-agent controllability, as in the equation below (CMO only model):

$$\mathrm{Mood}\left( t \right)=w_{0}+w_{1}\sum_{j=1}^{t} \gamma^{t-j}\mathrm{CMO}_{j}$$

This mood model was found to better explain the trial-by-trial mood in our task than all other models, including previously known models of momentary happiness [7] in the Bayesian model selection (PEP=1), and the CMO weight was significantly above 0 (mean(sd) of $w_{1}$: 2.382(1.633), t[102.00]=14.81, p<0.0001, CIs: 2.063 to 2.701, d=1.459, one-sample t-test, Fig. S4B; mean(sd) of $\gamma$: 0.517(0.201) and mean(sd) of $w_{0}$: 4.376(1.400)), showing that the intensity of the mood induced by the outcome is proportional to the inferred controllability. However, unlike the previous optimal model [7], our mood model did not include EV or RPE. We speculate that this difference between our study and Rutledge *et al*., 2014 might result from task differences. Unlike that previous study, our task involved another person who could be a controller, and the interaction between multi-agent controllability and reward might have become the most influential factor for one’s mood, while the influence of other factors, such as RPE and EV, decreased.

**Unidirectional Granger causality from the TPJ to the striatum**

We performed Granger causality analysis [9] on ROI time courses (TPJ and striatum). First, by following previous studies [10, 11], the trial-by-trial activity of the right striatum and the left TPJ was extracted in the following ways. First, the GLM design matrix was specified by using each trial as a separate event regressor (stick function). Each regressor was aligned to one second after the outcome (0 s and 2 s after outcome was also tested separately); this time point occurred prior to the time at which participants made causality decisions and mood ratings. In addition, regressors were convolved with a canonical hemodynamic response function, 24 nuisance regressors that were utilized in the whole-brain GLM analyses of the previous section were included, and low-frequency drifts were removed using a high-pass filter (128 s cutoff). We then used this GLM model to extract the BOLD signal of each trial. These BOLD signals 1 s after the outcome were averaged over the right striatum and the left TPJ ROIs, and the ROIs were the clusters found in the whole-brain GLM analyses of multi-agent controllability inference. Then, trial-by-trial BOLD signals 1 s after the outcome of two ROIs were used for Granger causality analysis.

It is called that time course Y G-causes time course X if the past time points of Y and X explain X better than the past of X alone. It is formulated by the log-likelihood ratio between the residual covariance matrix of the model that explains X by the past of X and Y and the residual covariance matrix of the model that explains X by the past of X alone [12]. Granger causality calculation was performed using the multivariate Granger causality toolbox (MVGC toolbox) in a default setting [12]. Granger causality analyses between the TPJ and striatum returned both Granger causality from the TPJ to the striatum and Granger causality from the striatum to the TPJ and their significance for each subject. To identify the significance of Granger causality at the group level, we used the permutation method to estimate the statistical significance of averaged Granger causality over subjects [13]. We generated 1000 surrogate Granger causalities for each ROI to make the permutation distribution of averaged Granger causality. At each iteration, surrogate time courses of both ROIs were generated by random permutation of original ROI time courses to estimate surrogate Granger causalities, which were then averaged over subjects. Because Granger causality is positively biased (Barnett, Seth, 2014), the significance value was calculated in a one-tailed fashion. Note that we did not consider the individual-level significance of Granger causality.

The results showed that there was a significant predictive causality from the TPJ to the striatum (p=0.013 in the permutation test, Fig. S7), while the reverse direction (striatum to TPJ) was not significant (p=0.184 in the permutation test, Fig. S7).

**Reward-dependent integration of self-likelihood on the signal indicating inferred controllability**

A relative influence of self-likelihood compared to that of other-likelihood on inferencing controllability was greater after reward than after loss. Therefore, if the TPJ and the striatum are involved in multi-agent inferred controllability, the relative influence of self-likelihood in making their neural activity in those regions might be greater after reward than after loss.

To test this hypothesis, we estimated the relative influence of self-likelihood on the trial-by-trial activity of the TPJ/striatum after reward and loss separately and compared the difference. Particularly, we used an equation similar to our multi-agent controllability inference model as follows:

$Activation_{TPJ/striatum}(t)=\frac{\mathrm{likelihood}_{\mathrm{self}}*exp(NB)}{\mathrm{likelihood}_{\mathrm{self}}*exp(NB)+\mathrm{likelihood}_{\mathrm{other}}}$ (14)

where NB is the neural bias parameter representing the relative weight of self-likelihood on the trial-by-trial activation of the region (TPJ and striatum). We computed the NB of each region after reward (NB_reward_) and loss (NB_loss_) separately and determined whether NB_reward_ was greater than NB_loss_ in both regions. Because the right-hand side of the above equation is between 0 and 1, the activity of each region was scaled to have values between 0 and 1 by applying the logistic function. Note that the TPJ and striatal activity used here were 1 s and 0 s after feedback, respectively.

This analysis showed a greater contribution of self-likelihood to neural activity after reward than after loss in both the striatum and TPJ (relative contribution of self-likelihood to striatal activity 0 s after the outcome: t[29.00]=3.18, p=0.0035, CIs: 0.176 to 0.812, d=0.580, one-sample t-test; TPJ activity 1 s after the outcome: t[29.00]=3.82, p=0.0007, CIs: 0.236 to 0.781, d=0.697, one-sample t-test).

**Multilevel mediation analyses on ROI time series**

We tested whether the striatum and TPJ mediate the influence of reward on vmPFC activity in the next trial using multilevel mediation analyses using the Mediation Toolbox (<http://wagerlab.colorado.edu/tools>) [14]. Mediation analysis tests whether the covariance between two variables (X and Y) can be explained by a mediator variable (M). A significant mediator is one whose inclusion as an intermediate variable in a path model of the effects of X on Y significantly affects the slope of the X-Y relationship. In these analyses, feedback (reward=1, loss=0) was the predictor variable (X), vmPFC activity of the next trial was the outcome variable (Y) and TPJ activity (1 s after the outcome) or striatal activity (0 s after the outcome) was the mediator variable (M). Trial-by-trial activities of each ROI were normalized. Similarly, in the second mediation analyses, feedback (reward=1, loss=0) was the predictor variable (X), the optimal choice was the outcome variable (Y=1 if the choice was optimal) and the TPJ or striatal activity was the mediator variable (M). Note that because positive bias was related to an increased optimal choice in the second half of the self-controllable block, we used these trials only in this second mediation analysis. Bootstrapping was used for significance testing of each path in the mediation analyses (path a, b, c’ and a x b) [14]. Subject-level path coefficients were randomly sampled with replacement 10000 times. Two-tailed p-values were calculated from the bootstrap confidence interval [14].

**TPJ mediates the effect of positive bias on optimal choice**

We tested whether reward-induced TPJ and striatal activity could result in an increase in actual behavioral optimality. Mediation analysis results showed that TPJ activity significantly mediated the effect of reward on the optimal choice in the next trial (mean(sd)=0.20(0.1), p=0.0444). However, striatal activity did not show such an effect (p>0.1).

**References**

1. Niv Y, Daniel R, Geana A, Gershman SJ, Leong YC, Radulescu A, et al. Reinforcement learning in multidimensional environments relies on attention mechanisms. Journal of Neuroscience. 2015;35(21):8145-57.

2. Mathys CD, Lomakina EI, Daunizeau J, Iglesias S, Brodersen KH, Friston KJ, et al. Uncertainty in perception and the Hierarchical Gaussian Filter. Frontiers in human neuroscience. 2014;8:825.

3. Huys QJ, Dayan P. A Bayesian formulation of behavioral control. Cognition. 2009;113(3):314-28.

4. Wilson RC, Collins AG. Ten simple rules for the computational modeling of behavioral data. eLife. 2019;8:e49547.

5. Stolz DS, Müller-Pinzler L, Krach S, Paulus FM. Internal control beliefs shape positive affect and associated neural dynamics during outcome valuation. Nature Communications. 2020;11(1):1-13.

6. Li P, Shen Y, Sui X, Chen C, Feng T, Li H, et al. The neural basis of responsibility attribution in decision-making. PloS one. 2013;8(11).

7. Rutledge RB, Skandali N, Dayan P, Dolan RJ. A computational and neural model of momentary subjective well-being. Proceedings of the National Academy of Sciences. 2014;111(33):12252-7.

8. Daunizeau J, Adam V, Rigoux L. VBA: a probabilistic treatment of nonlinear models for neurobiological and behavioural data. PLoS computational biology. 2014;10(1):e1003441.

9. Granger CW. Some recent development in a concept of causality. Journal of econometrics. 1988;39(1-2):199-211.

10. Kappes A, Harvey AH, Lohrenz T, Montague PR, Sharot T. Confirmation bias in the utilization of others’ opinion strength. Nature Neuroscience. 2020;23(1):130-7.

11. Fleming SM, Van Der Putten EJ, Daw ND. Neural mediators of changes of mind about perceptual decisions. Nature neuroscience. 2018;21(4):617-24.

12. Barnett L, Seth AK. The MVGC multivariate Granger causality toolbox: a new approach to Granger-causal inference. Journal of neuroscience methods. 2014;223:50-68.

13. Kim J, Park H-D, Kim KW, Shin DW, Lim S, Kwon H, et al. Sad faces increase the heartbeat-associated interoceptive information flow within the salience network: a MEG study. Scientific reports. 2019;9.

14. Woo C-W, Roy M, Buhle JT, Wager TD. Distinct brain systems mediate the effects of nociceptive input and self-regulation on pain. PLoS biology. 2015;13(1):e1002036.
